# Application of single-cell RNA sequencing in optimizing a combinatorial therapeutic strategy in metastatic renal cell carcinoma

**DOI:** 10.1186/s13059-016-0945-9

**Published:** 2016-04-29

**Authors:** Kyu-Tae Kim, Hye Won Lee, Hae-Ock Lee, Hye Jin Song, Da Eun Jeong, Sang Shin, Hyunho Kim, Yoojin Shin, Do-Hyun Nam, Byong Chang Jeong, David G. Kirsch, Kyeung Min Joo, Woong-Yang Park

**Affiliations:** Samsung Genome Institute, Samsung Medical Center, Seoul, South Korea; Institute for Future Medicine, Samsung Medical Center, Seoul, South Korea; Departments of Molecular Cell Biology, Sungkyunkwan University School of Medicine, Seoul, South Korea; Departments of Anatomy and Cell Biology, Sungkyunkwan University School of Medicine, Seoul, South Korea; Department of Health Sciences and Technology, Samsung Advanced Institute for Health Sciences & Technology, Sungkyunkwan University, Seoul, South Korea; School of Mechanical Engineering, Korea University, Seoul, South Korea; Departments of Neurosurgery, Samsung Medical Center, Sungkyunkwan University School of Medicine, Seoul, South Korea; Departments of Urology, Samsung Medical Center, Sungkyunkwan University School of Medicine, Seoul, South Korea; Departments of Pharmacology and Cancer Biology, Duke University Medical Center, Durham, NC USA; Departments of Radiation Oncology, Duke University Medical Center, Durham, NC USA

**Keywords:** Single cell analysis, Renal cell carcinoma, Patient-derived xenograft, Tumor heterogeneity, Drug response

## Abstract

**Background:**

Intratumoral heterogeneity hampers the success of marker-based anticancer treatment because the targeted therapy may eliminate a specific subpopulation of tumor cells while leaving others unharmed. Accordingly, a rational strategy minimizing survival of the drug-resistant subpopulation is essential to achieve long-term therapeutic efficacy.

**Results:**

Using single-cell RNA sequencing (RNA-seq), we examine the intratumoral heterogeneity of a pair of primary renal cell carcinoma and its lung metastasis. Activation of drug target pathways demonstrates considerable variability between the primary and metastatic sites, as well as among individual cancer cells within each site. Based on the prediction of multiple drug target pathway activation, we derive a combinatorial regimen co-targeting two mutually exclusive pathways for the metastatic cancer cells. This combinatorial strategy shows significant increase in the treatment efficacy over monotherapy in the experimental validation using patient-derived xenograft platforms *in vitro* and *in vivo*.

**Conclusions:**

Our findings demonstrate the investigational application of single-cell RNA-seq in the design of an anticancer regimen. The approach may overcome intratumoral heterogeneity which hampers the success of precision medicine.

**Electronic supplementary material:**

The online version of this article (doi:10.1186/s13059-016-0945-9) contains supplementary material, which is available to authorized users.

## Background

Clear cell renal cell carcinoma (ccRCC), the most prevalent type of sporadic kidney cancer, is often associated with malignant disease progression and poor therapeutic outcomes [1]. A major underlying genetic alteration in ccRCC is the von Hippel–Lindau (*VHL*) tumor-suppressor gene, whose deregulation stimulates an oncologic metabolic shift [2]. Signaling pathways involved in this metabolic shift have been proposed as potential therapeutic targets, including epidermal growth factor receptor (EGFR) [3], vascular endothelial growth factor (VEGFR) [4], or mammalian target of rapamycin (mTOR) pathways [5]. Although targeting these pathways significantly improved progression-free survival, the outgrowth of drug-resistant clones reduced the clinical efficacy and remains a clinical challenge that must be overcome [1].

Approximately 30 % of patients with renal cell carcinoma (RCC) are diagnosed with metastases [6]. Metastatic renal cell carcinoma (mRCC) evolves from primary RCC (pRCC) and harbors multiple subpopulations with distinct genomic [7, 8] and transcriptomic [9–11] features. Such intratumoral heterogeneity (ITH) is augmented by spatiotemporal tumor evolution [7, 8, 12]. The existence of disparate admixtures of cancer cells across mRCC and pRCC typically leads to significant differences in their sensitivity to therapies [13] and rare complete response to targeted molecular agents [1]. Therefore, independent investigation of mRCC and pRCC tumor tissues is essential to comprehend the intratumoral landscape within a patient and ultimately to achieve sustainable therapeutic benefit through a rational drug combination design. The magnitude of ITH in ccRCC has been discerned in detail by recent approaches of sequencing multiregional biopsied specimens [7, 8] or single cells [14]. However, experimental applications of the analyzed ITH signature to effectively eradicate tumor cells have not been extensively investigated.

Accurate identification of disease-causing sequence variants and driving pathways is essential to minimize drug resistance or tumor relapse with targeted therapeutics [15]. Genomic mutations, however, may have limited functional significance as druggable targets, according to previous findings that drug responses can be widely different even in genetically homogeneous cancer cell lines [16, 17]. A systematic assessment from the National Cancer Institute and the Dialogue on Reverse Engineering Assessment and Methods (NCI-DREAM) project has shown that gene expression profiling has the best predictive power among independent profiling platforms, with increasing power upon data integration [18]. In the prediction of drug sensitivity in cancer cells, transcriptome profiling can enhance our understanding of how the cellular mechanism is functionally perturbed in response to drug treatment [19]. Moreover, considering that single targeting agents may eliminate a certain subpopulation of tumor cells while leaving others unharmed, it is necessary to analyze the tumor transcriptome at high resolution to detect drug-resistant clones that may be concealed within ITH. Gene expression profiling at a single-cell resolution may enable modeling of functional heterogeneity and identification of subpopulations with specific drug responsive signatures.

Here, we used single-cell RNA sequencing (scRNA-seq) not only to elucidate transcriptional heterogeneity during the metastatic progression, but also to design an optimized combination of targeted agents against metastatic RCC. On the basis of single-cell transcriptome analysis, identification of cellular subpopulations with distinct activation status of signaling pathways allowed us to postulate a combinational therapeutic regimen with an increased likelihood of covering all potentially targetable cancer cells.

## Results

### Establishment of patient-derived xenografts from paired pRCC and mRCC

**Fig. 1:**
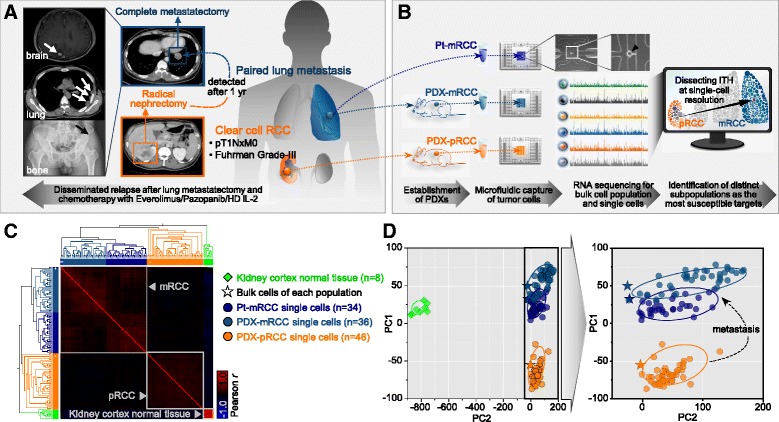
Profiling transcriptome of paired pRCC and mRCC at single-cell resolution. **a** Brief description of clinical course in a patient with metastatic RCC. **b** *Schematic* of scRNA-seq experiments from establishment of the patient-derived xenograft model to discovery of targetable subpopulations. **c** Hierarchical clustering *heatmap* and *dendrogram* based on inter-correlation of centroid global expression profiles across kidney cortex normal, bulk cells of each population, and single cells using Euclidean distance metric and average linkage. **d** Principal component analysis (PCA) of single-cell-resolved gene expression profiles based on the first two principal components. *Ellipses* represent 95 % confidence around each group

The patient exhibited intrinsic refractoriness to sequential conventional therapies, including pazopanib, everolimus, and high-dose interleukin-2, resulting in rapid multiorgan dissemination of cancer cells after pulmonary metastectomy (Fig. 1a). To verify the data-driven prediction of putative therapeutic targets we used paired pRCC and mRCC patient-derived xenografts (PDX) for RCC, which enabled us to understand the ITH at the cellular level [20] with the benefits of recapitulating the molecular, genetic, and histopathologic heterogeneity of the parental tumors [21–23]. The parental tumors and PDXs shared histopathologic characteristics, including subtype-specific morphologic features and differentiation status (Additional file 1: Figure S1A). The cancer cell fractions were highly enriched in the PDX models (Additional file 1: Figure S1B), as previously observed [24]. Although there were discordant somatic single-nucleotide variants (SSNVs) between parental tumors and PDX samples (Additional file 1: Figure S1C) from whole-exome sequencing (WES) analysis, genes that are frequently mutated in ccRCCs [25] persisted in all tumor samples (Additional file 1: Figure S1D). Copy number variations detected from array comparative genomic hybridization (aCGH) analysis in parental tumors were also preserved in PDX tumors (Additional file 1: Figure S1E and F). Together, these data indicate that human tumors from pRCC and mRCC were propagated through xenograft engraftments with consistent features of histopathology and genomic landscape.

### Evolutionary genomic trajectories during tumor progression and metastasis

In recognition of that genomic features were consistently propagated with higher cancer cell fraction (~100 %) through xenograft passaging (Additional file 1: Figure S1), we investigated genomic architectures in the pRCC and mRCC tumors from PDX samples to understand the clonal evolution associated with the spatiotemporal tumor progression. WES analysis of primary and paired metastatic samples revealed that 23.5 % of SSNVs were shared (Additional file 2: Figure S2A). In particular, a *VHL* D121G mutation was found in both samples with high allele frequencies (~1.0, Additional file 2: Figure S2A and Additional file 3: Table S1), suggesting that it might be a founder event in tumor evolution [7, 8]. Variant allele frequencies (VAF) of the shared SSNVs were typically higher than those of SSNVs exclusively observed in mRCC (38 %) or pRCC (38.6 %) (Additional file 2: Figure S2A). Discordant SSNVs in mRCC and pRCC might result from the gradual increase in point mutations and clonal selection with tumor evolution, as previously reported [7, 8]. In contrast, somatic copy number alterations (SCNAs) in mRCC were similar to those in pRCC (Additional file 2: Figure S2B), with 5q amplifications detected only in pRCC (Additional file 2: Figure S2C). Integrated analyses of WES and aCGH to infer evolutionary trajectories showed that major clones harboring driver mutations were shared at high cellular frequencies, whereas minor subclones were enriched in mRCC (Additional file 4: Figure S3A, B and Additional file 5: Table S2). Overall, the RCC of our patient showed a complex non-linear branching clonal evolution (Additional file 4: Figure S3C) that may become the basis of intratumoral diversity [7, 8, 12]. The genetic complexities might also result in molecular and functional differences between pRCC and mRCC despite their clonal origin, as previously reported [9–11].

### Single-cell RNA sequencing and quality assessment for expression profiling

To model the functional heterogeneity and to identify specific subpopulations that are phenotypically relevant to drug responses, we used scRNA-seq to profile single cells from the parental mRCC and PDX mRCC and pRCC (Fig. 1b and see “Methods”). After filtering out poor-quality cells, a total of 116 tumor cells from the parental mRCC (n = 34), PDX-mRCC (n = 36), and PDX-pRCC (n = 46) were used in subsequent analyses (Additional file 6: Figure S4 and Additional file 7: Table S3). When compared to the normal kidney cortex, single cancer cells had much more variable gene expression as shown by the high coefficient of variation for averaged gene expression (Additional file 8: Figure S5A). Nonetheless, housekeeping genes, including glyceraldehyde 3-phosphate dehydrogenase (*GAPDH*) and beta-actin (*ACTB*), were stably expressed across single cells with low variation within (Additional file 8: Figure S5A) and across (Additional file 8: Figure S5B) cell populations, suggesting functional significance of the gene expression heterogeneity.

Compared to the normal kidney cortex, parental and PDX cancer cells demonstrated low stromal and high ccRCC gene expression signatures (Additional file 9: Figure S6). Interestingly, the global expression profiles of patient-mRCC and PDX-mRCC single-cell samples were more closely related than those of PDX-pRCC samples (Fig. 1c and Additional file 10: Figure S7). Principal component analysis (PCA) across all samples showed three distinctive main clusters (Fig. 1d); in addition to a cluster of normal kidney cortex, a cluster of parental mRCC cells and PDX samples were separated from a cluster of PDX-pRCC samples. As identified in unsupervised clustering of global expression across samples (Fig. 1c), we found the averaged expression of single cells correlated well with that of their bulk cell population samples (Additional file 10: Figure S7B). Furthermore, multiple regression analysis on the transcriptomes of different sized pools of single cells to those of bulk cell population samples showed better representation of the bulk cell population with increasing number of single cells (Additional file 10: Figure S7C). Similarly, in the PCA plot, bulk cell population samples almost matched single cells (Fig. 1d). As the quality assessments and unsupervised clustering analyses demonstrate the reliability of our data, we further explored the single-cell transcriptomes.

### Metastatic and aggressive molecular signatures in mRCC transcriptomes

Consistent with the previous observation that PDX cells reflect their parental tumors with rare contamination of normal stromal cells [24], we verified discrete global expression profiles of single RCC cells compared with normal kidney expression profiles (Fig. 1c, d, Additional file 9: Figure S6, and Additional file 10: Figure S7). The stromal signature of parental bulk mRCC was higher than in other tumor samples (Additional file 9: Figure S6A), concordant with genomic analysis showing a smaller tumor portion in parental mRCC than PDX samples (Additional file 4: Figure S3B). By comparison, single cells from the parental mRCC all showed low stromal signatures (Additional file 9: Figure S6A) and stably higher ccRCC signatures (Additional file 9: Figure S6B), suggesting selective capture of tumor cells from the bulk cell populations. This might be explained by the experimental approach of using large size (17–25 μm) microfluidic chips to capture single cells based on the histopathologic properties of the RCC tumor cells (Fuhrman Grade-III, average cell size ~20 μm) [26] to reduce the likelihood of capturing stromal cells.

**Fig. 2:**
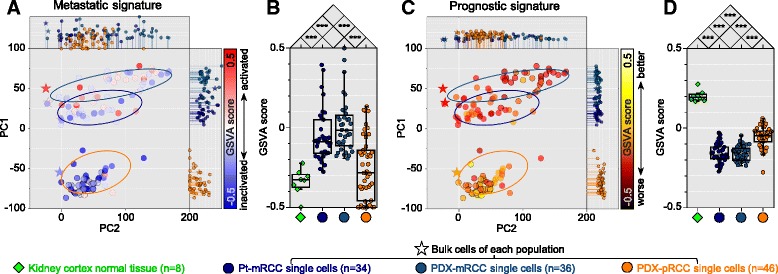
Evaluation of expression signatures associated with metastatic RCC across single cells. **a**–**d** Gene set activation analysis for the metastatic signature (**a** and **b**) and clear cell RCC prognostic signature (**c** and **d**). **a**, **c** Positions of each *dot* and *ellipse* in Fig. 1d were fixed, and then each *dot* was colored (*main panel*) and indicated as *drop lines* (*top and right panels*) according to the estimated status for the given signatures. Gene Set Variation Analysis (GSVA) scores were normalized to normal kidney tissue expression profiles. **b**, **d** *Boxplots* show overall reciprocal differences in the expression signatures across normal kidney cortex, bulk cells of each population, and single cells. *Boxes* show 25th to 75th percentile with 10th and 90th percentile whiskers. *P <0.05, **P <0.01, ***P <0.001, two-tailed Student’s *t*-test

We then sought to identify distinct molecular signatures between mRCC and pRCC tumors. In recognition of the critical role of epithelial–mesenchymal transition (EMT) in promoting metastasis [27, 28], we first examined the “EMT-induced” signature. In the virtual space of the PCA plot (Fig. 1d), the EMT-induced signature was enriched both in patient-mRCC and PDX-mRCC samples compared to PDX-pRCC samples (Additional file 11: Figure S8A) with statistical significance (Additional file 11: Figure S8B). Compared to the parental pRCC tumor, mRCC samples also showed elevated metastatic signatures (Fig. 2a and b). Because metastatic tumors tend to have worse prognostic properties and poor survival outcomes [29], we evaluated the “tumor aggressiveness” using prognostic markers that classify patients according to their survival rates [25]. Similar to the EMT-induced signature, we observed worse prognostic marker expression in mRCC cells, suggestive of tumor aggressiveness (Fig. 2c, d). From biological enrichment analysis with differentially expressed genes between pRCC cells and mRCC cells, Gene Ontology terms supporting tumor aggressiveness, such as “regulation of cell proliferation,” “regulation of cell death,” or “regulation of response to stress,” were also enriched in mRCC cells (Additional file 12: Figure S9). Collectively, these results demonstrate distinct gene expression properties of mRCC cells, with enhanced metastatic and aggressiveness signatures compared to pRCC cells.

### Prediction of activation of drug target pathways and sensitivity to drug responses

**Fig. 3:**
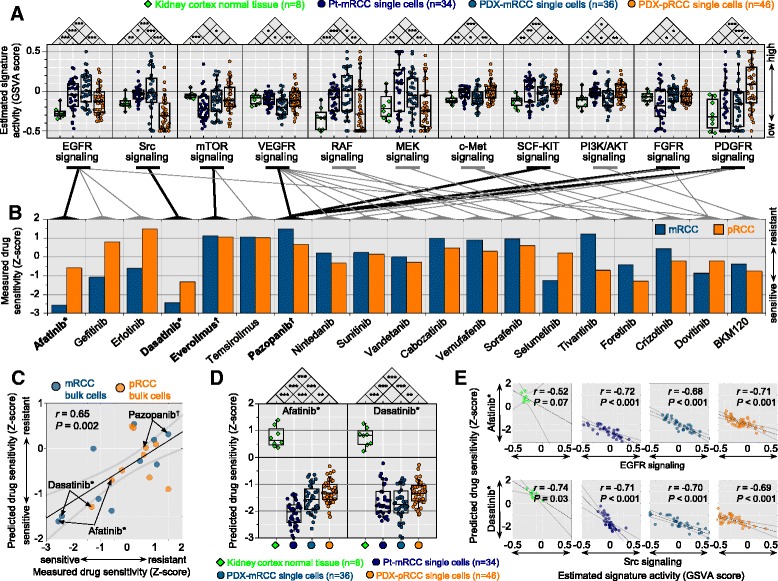
Identification of targetable signaling pathways by transcriptome profiling and drug screening. **a** Quantitative estimation of the activation status of targetable signaling pathways across single cells. *Boxplots* demonstrate overall reciprocal differences in expression signatures across normal kidney cortex, bulk cells of each population, and single cells. **b** Measured drug response profiles of pRCC and mRCC cells, matched to the targetable signaling pathways. Sensitivities of cells to various targeted drugs were determined based on the half-maximal inhibitory concentration (IC_50_), and transformed to Z-scores. Afatinib and dasatinib were selected as the most effective drugs against mRCC cells (denoted as *) whereas everolimus and pazopanib (denoted as †) showed no effects, which is consistent with clinical findings. **c**–**e** Drug sensitivity was predicted by the ridge regression model using a training set of publicly available cancer cell line expression data with each of the measured IC_50_ data. Estimated values were transformed to Z-scores across samples. **c** Significant correlation of predicted drug sensitivity with measured sensitivity in **b**. **d** Comparison of the predicted drug sensitivity of afatinib and dasatinib between populations. **e** For the selected drugs afatinib and dasatinib, there was a significant correlation between predicted drug sensitivity (Z-scores) and activation status (GSVA scores) of the relevant targeted pathways. **c**, **e** Linear regression was applied to estimate Pearson’s correlation coefficient (*r*), with 95 % confidence as shown by *thicker light gray curves*. The statistical significance of the regression was determined by one-way ANOVA test. **a**, **d** *Boxes* show 25th to 75th percentile with 10th and 90th percentile whiskers. *P <0.05, **P <0.01, ***P <0.001, two-tailed Student’s *t*-test

Distinct gene expression profiles in pRCC and mRCC tumor cells suggest divergent tumor cell behavior, including altered drug responses. Using predefined gene sets involved in drug target pathways, we estimated the relative activation status of the drug sensitivity signatures across PDX-mRCC and PDX-pRCC cells. Many drug target pathways were differentially regulated in the two PDX tumor cell groups (Fig. 3a and Additional file 13: Figure S10), suggesting differential drug sensitivity. We subsequently screened the PDX-mRCC and PDX-pRCC cells with a panel of targeted agents (Fig. 3b and Additional file 14: Table S4). In repeated measures analysis for drug sensitivity, we observed nearly identical measurements in duplicate with a high statistical power (Additional file 15: Figure S11). PDX-mRCC cells showed significantly higher expression of genes in the EGFR, Src, and BRAF/MEK pathways compared to PDX-pRCC cells, suggesting these pathways as mRCC-specific druggable targets. The PDX-mRCC cells showed ample responses to agents targeting EGFR (gefitinib, erlotinib, and afatinib), Src (dasatinib), and BRAF/MEK (selumetinib), substantiating the prediction. On the other hand, gene expression and activation scores in c-Met and PI3K/AKT pathways were significantly higher in PDX-pRCC cells. Concordantly, PDX-pRCC cells were more sensitive to agents targeting c-Met (tivantinib, foretinib, and crizotinib) and PI3K (BKM120) than PDX-mRCC cells.

To refine the predictive power of signaling pathway activation for drug sensitivity, we built a ridge regression model [30] using public gene expression profiles and drug sensitivity data as a training set [31]. For the PDX-mRCC and pRCC bulk cell population samples, we found strong positive correlations between predicted and measured drug sensitivity (Fig. 3c). Drug sensitivity was also estimated in all single cancer cells and in normal kidney cortex as a control; these data suggested afatinib and dasatinib sensitivity of pRCC cells (Fig. 3d), similar to the predicted and measured drug sensitivity of bulk cell populations (Fig. 3b). The prediction of drug sensitivity by the ridge regression model was in agreement with the estimation of drug target pathway activation across single cells (Fig. 3e and Additional file 16: Figure S12). Interestingly, pazopanib and everolimus, which showed no clinical benefit in this patient (Fig. 1a), had minor effects on the viability of PDX-pRCC and PDX-mRCC cells (Fig. 3b and Additional file 17: Figure S13) and signaling pathway activation for their targets mTOR and VEGFR also remained low (Fig. 3a and Additional file 13: Figure S10). Although drug sensitivity of cancer cells was predicted to be higher than that of normal kidney cortex for all of the anticancer agents, the difference in pazopanib sensitivity was small (Additional file 16: Figure S12A). Overall, drug sensitivity predictions showed significant correlations with corresponding signaling pathway activation (Additional file 16: Figure S12B). We estimated the activation status of signaling pathways and drug sensitivity and demonstrated concordance between the predicted signatures and the measured data. These results suggest that molecular targeted therapies can be designed on the basis of prediction signatures obtained from RNA-seq.

### Heterogeneity in drug response signatures within a tumor

**Fig. 4:**
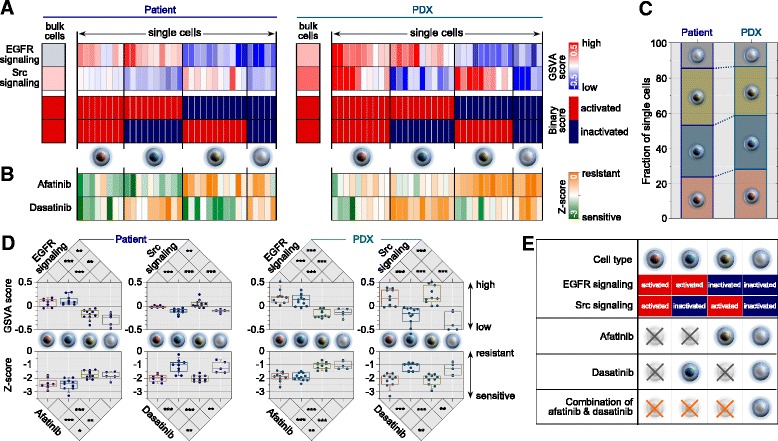
Dissection of single-cell subpopulations according to distinctive activation status of EGFR and Src pathways. **a**, **b** Single cells were manually ordered by categorization into four groups according to their activation status as shown in **e**. **a** *Heatmaps* showing the relative (*upper*) and binary (*lower*) activation status for EGFR and Src pathways in mRCC cells from parental and PDX tumors. **b** Predicted drug sensitivity of afatinib and dasatinib matched to **a**. **c** Comparison of the fraction of categorized single cells between parental and PDX tumors. **d** Differences in the estimated pathways with predicted drug sensitivity among the four classified groups. *Boxes* show 25th to 75th percentile with 10th and 90th percentile whiskers. *P <0.05, **P <0.01, ***P <0.001, two-tailed Student’s *t*-test. **e** Strategic combination therapy using afatinib and dasatinib to target the EGFR and Src signaling pathways, respectively

Dysregulation of EGFR [32–34] and Src/FAK [35, 36] pathways plays a critical role in the tumorigenesis and metastatic progression of RCCs. The anticancer agents afatinib [37] and dasatinib [38, 39], targeting EGFR and Src signaling, respectively, have shown efficacy in the treatment of metastatic RCC. In drug sensitivity prediction and screens, PDX-mRCC cells showed the highest sensitivity to afatinib and dasatinib among the various drugs tested (Fig. 3b). At the single-cell level, PDX-mRCC showed extensive heterogeneity in the activation status of the EGFR and Src pathways (Fig. 3a and Additional file 13: Figure S10). This heterogeneous cellular pattern enabled us to classify cells according to the binary activation status of EGFR and Src signaling pathways (Fig. 4a), which was corroborated by the drug sensitivity prediction (Fig. 4b and Additional file 18: Figure S14). Notably, the classified cellular fractions were similar between the parental mRCC and PDX-mRCC (Fig. 4c), suggesting that heterogeneity in drug sensitivities for the PDX model reflects that of the parental tumor.

The heterogeneous drug sensitivity prediction for individual tumor cells suggests the presence of tumor subpopulations with distinct signaling pathway activation and drug sensitivity. Based on the prediction results, we identified four mRCC subpopulations with corresponding signaling pathway activation for EGFR and Src pathways (both active, only EGFR active, only Src active, both inactive; Fig. 4). Only a fraction of cells (23.5 % in parental mRCC; 27.8 % in PDX-mRCC) with activated status for both in EGFR and Src signaling pathways were predicted to be sensitive to both afatinib and dasatinib. A population with inactivated status in both signaling pathways (14.7 % in parental mRCC; 13.9 % in PDX-mRCC) is unlikely to respond to afatinib or dasatinib. The largest proportion of cells (61.8 % in parental mRCC; 58.3 % in PDX-mRCC) had signaling pathway activation in either of the two pathways, suggesting a mutually exclusive response to the corresponding drugs. This classification suggests that co-targeting of EGFR and Src with a combination of afatinib and dasatinib would be an efficient therapeutic strategy without redundancy and with additive antitumor effects (Fig. 4e).

### Evaluation of the single-cell analysis-driven therapeutic strategy

**Fig. 5:**
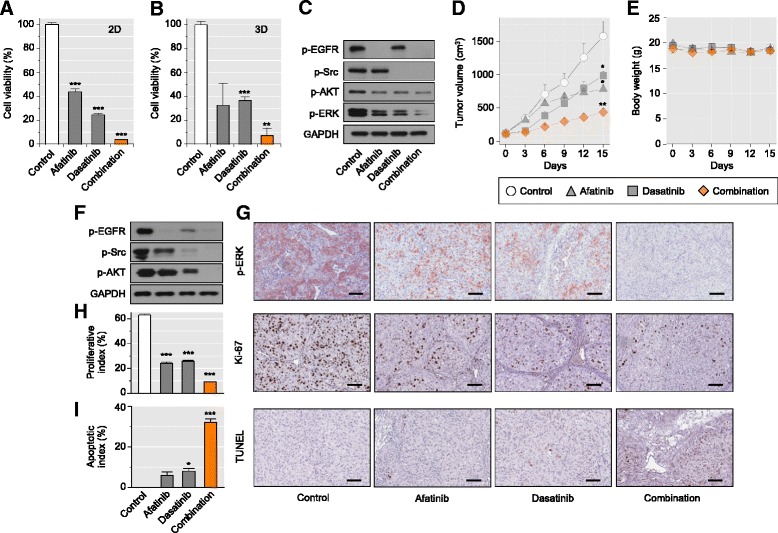
Effective combinatorial strategy targeting heterogeneous subclones in mRCC. **a**, **b** Combined effects of afatinib and dasatinib on viability of mRCC cells were analyzed 6 days after treatment under 2D non-adherent culture conditions **a** or 3D ECM scaffold culture system **b**. **c** Effects of afatinib and dasatinib combination therapy on EGFR and Src downstream pathways were validated by immunoblotting. Cells were incubated with 0.5 μM afatinib and/or dasatinib for 1 h. GAPDH = loading control. Cells that were mock-treated with 0.5 μM DMSO served as negative controls. Error bar = SEM (n = 3 for each group). **P <0.01, ***P <0.001. **d**–**i** Superior antitumor efficacy of combinations of afatinib and dasatinib in mRCC subcutaneous xenografts. **d** Mice bearing mRCC tumors (=5 mice/group) were p.o. dosed with afatinib (each at 20 mg/kg) or i.p. administered dasatinib (each at 15 mg/kg), either alone or in combination, on a daily dosing regimen for up to 15 days. Growth curves based on tumor volumes are shown as the mean ± SEM for each time point. *P <0.05, **P <0.01. **e** Changes in body weight of mice treated with afatinib and/or dasatinib. Body weight was measured on the indicated days. Data show mean ± SEM. Tumor tissues from each group were harvested on day 15 and subjected to immunoblot analysis with the indicated antibodies to detect p-EGFR, p-Src, and p-AKT (**f**; GAPDH = loading control), or immunostained with anti-p-ERK antibody **g**. Proliferation and apoptotic rates in each group were determined by Ki-67 immunostaining **h** and TUNEL assay **i**. Results are presented as mean values ± SEM. *P <0.05, ***P <0.001. Scale bars = 100 μm

Having recognized the existence of distinct subpopulations with differential drug sensitivities, we tested the efficacy of co-targeting EGFR and Src signaling pathways in PDX-mRCC cells. On the basis of transcriptome profiling and high-throughput drug screening (Fig. 3), we first measured the growth inhibitory effects of afatinib and dasatinib (Fig. 5a) in a two-dimensional (2D) culture system. Notably, compared to single drug treatment, the combination therapy more effectively suppressed the viability of mRCC cells (Fig. 5a). The efficiency of the combinatorial drug treatment was also examined in the spheroid formation model, which is a well-characterized three-dimensional (3D) culture and screening model with the advantages of simplicity, reproducibility, and similarity to physiological tissues compared with other methods involving extracellular matrix scaffolds and hydrogel systems [40, 41]. Consistent with the results obtained from the 2D system, we observed a superior effect of the combination therapy in the 3D system (Fig. 5b). Co-treatment with afatinib and dasatinib caused complete abrogation of EGFR and Src activity and more efficient inhibition of downstream AKT and ERK phosphorylation in PDX-mRCC cells than that achieved by monotherapy (Fig. 5c).

Finally, we tested the in vivo performance of the combinatorial drug treatment using the subcutaneous xenograft model of mRCC. As single agents, afatinib (20 mg/kg) or dasatinib (15 mg/kg) had modest inhibitory effects on tumor growth (afatinib, 55 %; dasatinib, 40 %; Fig. 5d). Despite the partial treatment effect, the tumor cells continued to grow and none of the tumor xenografts showed complete response. In combination, afatinib and dasatinib showed a significantly enhanced antitumor effect, inhibiting tumor growth by 78 % (Fig. 5d). Single-agent and combination treatment protocols were well tolerated by the mice, with no weight loss or other signs of acute or delayed toxicity (Fig. 5e). The drug effects were confirmed at the molecular level by complete inhibition of AKT (Fig. 5f) and ERK (Fig. 5g) phosphorylation. Together, co-targeting functionally distinct subpopulations in mRCC PDX cells that were identified by single-cell transcriptome analyses significantly improved treatment outcomes in both in vitro and in vivo investigational models.

## Discussion

The evolution of multiple tumor subclones during tumor progression and metastasis generates intratumoral heterogeneity, which plays a role in intrinsic and acquired treatment resistance to molecular targeted therapies. In mRCC, durable complete responses are rarely achieved with conventional targeted molecular agents [1]. Furthermore, primary tumors and their corresponding metastases frequently show significant differences in therapeutic responses [13], suggesting that biopsies should be taken upon metastatic relapse for molecular profiling analysis to inform selection of salvage therapies. To date, most molecular profiling has been performed in the primary tumors of RCC patients and therefore failed to recapitulate the metastatic population. To overcome such clinical challenges, therapeutic strategies that efficiently target heterogeneous tumor subpopulations in mRCC must be developed.

In this report, we introduce scRNA-seq as a promising strategy that allows high-resolution analyses of the intratumoral landscape for tumor transcriptomes [42] and optimization of targeted treatment regimens against metastatic RCC. In our model case, we found that mRCC diverged from pRCC both at genomic and transcriptomic levels, manifesting distinctive genetic aberrations and drug target pathway activation. We combined comprehensive single-cell analyses with high-throughput drug screening in the PDX model and, based on these data, suggested a combinatorial treatment regimen targeting both EGFR and Src as the most efficient therapeutic option. In recognition of that drug responses are diverse in the pathologically identical tumors and even in the genetically homogeneous cancer cells [16, 17], profiling tumor transcriptome at the single-cell resolution can enable us to dissect inherent complex heterogeneous cell populations and to discern which cell would be different in drug responses affecting the ultimate outcomes that are covered under stochastic averaged signals in the bulk cell measurement. This strategy can be applied to other investigational models and eventually to patients with refractory cancer. Since higher engraftment rates are associated with more clinically aggressive tumors such as metastatic or treatment-refractory cases [43], surgically removed metastases or biopsy metastasis samples might extend the application of PDXs to advanced RCCs in terms of technical feasibility and unmet clinical needs.

Clonal genetic events in the metastases can be demonstrated for restricted subclones of the primary tumor, suggesting that only rare cells within the primary tumor have the ability to metastasize [44]. The parallel progression model proposes independent evolution of early disseminates in distant sites, eventually leading to significant divergence between the primary and metastatic tumors [45]. However, it remains largely unknown whether mRCCs also have initial ITH or whether the development of mRCCs is due to early dissemination or late diagnosis. Integrative analyses of PDXs derived from pRCC and distant metastasis to the lung from a single patient allowed us to infer which clonal and subclonal alterations contributed to tumor progression and spread to the metastatic lesion. The genetic differences between the pRCC and mRCC suggest highly complex non-linear branching clonal evolution as the basis for molecular and functional diversity, consistent with previous studies [7, 8]. Moreover, key pathways that are targeted by clinically available drugs showed distinct expression patterns between pRCC and mRCC. Most driver aberrations and actionable driver mutations that have therapeutic implications were located on the branches, suggesting that distinct intratumoral subclones had acquired different functional characteristics [8]. The heterogeneity of metastatic cancer may underlie its poor responsiveness to therapy and explain why biomarkers of prognosis or therapy responsiveness measured exclusively from primary tumors give a restricted view of the biological properties of metastatic cancer [46].

Acquired resistance to therapy and disease progression can be due in part to intrinsic heterogeneity, including genetic, epigenetic, and biological properties of cancer cells that contribute to oncogenic activity. Thus, instead of single-agent therapy a rational approach that targets multiple subpopulations of tumor cells with a combination of non-cross-resistant drugs characterized by different mechanisms of action and non-overlapping profiles of toxicity will be necessary for long-lasting inhibition of highly heterogeneous mRCC [7, 8]. Knowledge of molecular alterations and features of tumors and the identification of mechanisms of tumor resistance provide the opportunity to test novel rationally designed drug combinations. Recent technological advances in single-cell sequencing have facilitated the paradigm shift in our understanding of the cancer ecosystem from the averaged signal of a complex tumor mass to the sum of distinctive signals in individual cells [42]. Unique cellular behaviors reflecting oncogenic signatures have been extensively scrutinized by profiling transcriptomes from individual cells in various types of cancers [24, 47–49]. In the light of the precedent approaches for deconvolving heterogeneous cell populations and identifying specific cell types that are generally masked in bulk cell profiling, we could understand better the biologically relevant composition of cancer cells and their functional modulation within the tumor. Importantly, in this study such dissected intratumoral landscape enabled us to elicit the most effective drug combination of putting all the potentially targetable cancer cells together to be killed.

## Conclusion

Using scRNA-seq, we could examine the heterogeneous drug target pathway activations at the single-cell level in a refractory mRCC patient. Distinct features of intratumoral expression variability across mRCC single cells that were masked in the bulk measurement prompted us to test the co-targeting strategy for the most vulnerable two signaling pathways with increased likelihood of complete response. Indeed, we observed significantly better treatment effects of the targeted combination therapy on mRCC-derived xenograft platforms in vitro and in vivo than monotherapies. Our findings described here will have a profound impact on translational research to overcome ITH-derived resistance and avoid ineffectual or unnecessary treatments. Although we could not analyze clinical response to our combination strategy because of rapid deterioration of the patient, we stress the utility and validity of single-cell transcriptome profiling in patients with refractory cancer for the design of personalized therapeutic strategies. In summary, the realization of the advantages in dissecting heterogeneous drug target pathway activations by scRNA-seq analysis will have significant clinical utility for the design of tailored combination therapy against highly heterogeneous tumors.

## Methods

### Ethics

This study was carried out in accordance with the principles of the Declaration of Helsinki, and was approved by The Samsung Medical Center (Seoul, Korea) Institutional Review Board (IRB) (no. 2010-04-004). Participants in this study gave written informed consent for the research and publication of the results. Animal experiments were conducted in accordance with the Institute for Laboratory Animal Research Guide for the Care and Use of Laboratory Animals and the following protocols were approved by the IRB at the Samsung Medical Center (Seoul, Korea) (No. 20131217002). Animal care and handling was conducted in accordance with the National Institute of Health Guide for the Care and Use of Laboratory Animals (NIH publication no.80-23, revised 1978).

### Sample selection and clinical characteristics of the patient

PDXs were established using surgically resected matched pRCC (pT1Nx; Fuhrman Grade 3) and paired lung metastasis from a 43-year-old man with ccRCC who experienced solitary lung metastasis 1 year after radical nephrectomy (IRB number 2010-04-004). He showed signs of rapid tumor dissemination to the bone, lung, pleura, and brain despite multiple salvage regimens, including pazopanib, everolimus, and high-dose interleukin (IL)-2, and finally died 16 months after complete metastatectomy as a result of rapid tumor progression. Serial 5-μm sections from each formalin-fixed paraffin-embedded block were processed for hematoxylin and eosin (H&E) staining and examined by a specialized pathologist.

### Establishment of orthotopic PDXs

Athymic nude mice were obtained from Orient Bio (Seoul, Korea). Fresh tumor tissue was obtained from pRCC tissue of the patient by surgical excision under sterile conditions and matched fresh tumor tissue was taken from the mRCC. Each biopsied parental tumor mass was chopped into fragments, and was frozen or placed in formalin and embedded in paraffin for later analyses. A blood pellet was used for extraction of germline DNA. Fresh tumor tissue was stored on ice in Hank’s Balanced Salt Solution (Gibco, Grand Island, NY, USA) supplemented with penicillin/streptomycin (Gibco) for transport. For transplantation, 6- to 8-week-old NOD scid gamma mice were anesthetized with 100 mg/kg ketamine and 10 mg/kg xylazine. Primary tumor and paired lung metastasis samples minced into approximately 1-mm^3^ fragments in high-concentration Matrigel TM Basement Membrane Matrix (BD Biosciences, Franklin Lakes, NJ, USA) were directly implanted into the subrenal capsule (n = 4–5 for each tumor sample). For subrenal capsule implantation, xenograft tumor engraftment was defined as a palpable mass of >1 mm in diameter with pathologic confirmation. When the resulting tumors grew, each tumor (F1 generation) was resected as the primary tumor, divided, and passaged into five mice (F2 generation). The PDX tumors were harvested and divided into three samples for generation of second in vivo passage xenograft tumors, DNA/RNA extraction, and histopathologic examination. The origin of each xenograft was validated by short tandem repeat DNA fingerprinting. The process was repeated to produce subsequent generations via subcutaneous implantation in BALB/c nude mice to expand xenograft numbers.

### Whole-exome sequencing

We extracted genomic DNA from patient-derived tumor samples using the QIAamp DNA Minikit (Qiagen, Hilden, Germany) and from matched blood using the QIAamp DNA Blood kit (Qiagen). Purified DNA was sheared to an average size of 150 bp in a Covaris Adaptive Focused Acoustics™ (AFA) sonication device (S2, Covaris, Inc., Woburn, MA, USA) and indexed with unique barcode tags using PCR. Prepared libraries were assessed for quality and quantity using a Qubit 2.0 Fluorometer (Life Technologies, Carlsbad, CA, USA), 2100 Bioanalyzer (Agilent Inc., Palo Alto, CA, USA), and Mx3005P qPCR (Agilent Technologies, Inc). Exomes were targeted using the SureSelect XT Human All Exon V5 kit (Agilent). Samples were multiplexed and flow-cell clusters were created using the TruSeq Rapid Cluster kit and TruSeq Rapid SBS kit (Illumina, San Diego, CA, USA). Captured exomes were sequenced using the Illumina HiSeq 2500 platform, and paired-end 100-bp sequence data were generated. Sequencing reads that only mapped to the mouse genome reference (mm10) were filtered out.

### Array comparative genomic hybridization

Purified DNA from patient-derived tumor samples was labeled with Cy5-dUTP following the Agilent Oligonucleotide Array-Based CGH for Genomic DNA Analysis protocol (Ver-7.3, Agilent). Cy5-labeled DNA was quantified together with reference DNA samples labeled with Cy3-dUTP to determine the DNA concentration using an ND-1000 Spectrophotometer (NanoDrop, Wilmington, DE, USA). Labeled test and reference samples were then hybridized to SurePrint G3 Human CGH 4 × 180 K Microarrays (Agilent) according to the manufacturer’s standard protocol. The dual-colored fluorescence signals were scanned using the Agilent Microarray Scanner and translated to log10 ratios using Feature Extraction software (Ver-11.0.1.1, Agilent).

### Identification of single-nucleotide variants and copy number variants

Using the WES data, generated reads were aligned to the human genome reference hg19 using the Burrows-Wheeler Aligner [50] after removing duplicate reads, followed by implementation of the data-processing modules using the Genome Analysis Toolkit [51]. Somatic SNVs were identified by Bayesian statistical analysis of bases and their qualities in the given tumor and paired normal BAM files at the genomic locus using the MuTect algorithm [52]. Called variants were reviewed manually using the Integrative Genomic Viewer [53], and considered only within chromosomes 1–22 and X. Mutations were annotated using the SnpEff [54] package.

Based on the CGH data, extracted signals were normalized to log2 ratios using the limma package [55]. To detect significant breakpoints across thousands of probe-derived signals, we applied the circular binary segmentation (CBS) algorithm using the DNAcopy package [56]. After smoothing the data to detect outliers within autosomal chromosomes, aberrant segments were determined applying the significance level of 1.0E-04 to accept change-points based on a maximum t-statistic. We classified the segmented results into copy losses when the log2 ratios were lower than −0.25 and copy gains when these were greater than 0.25. Considering sample-specific tumor purity and ploidy, somatic copy number alterations (SCNA) were adjusted by implementing the ABSOLUTE algorithm [57]. To compare the SCNA patterns across samples, segment values were averaged with 1-kb binning along the chromosomes.

### Clustering of genomic clones

To determine the subclonal structure between primary RCC and paired lung metastasis, we adopted the PyClone algorithm [58] that computes the cellular prevalence of mutations and clusters these mutations based on a hierarchical Bayes statistical model. Mutational information, including somatic SNVs called from the deep exomes and absolute copy-number changes corresponding to SNV regions, was prepared for use with PyClone. The cellular prevalence for each mutation was estimated using a beta-binomial model by setting the number of Markov chain Monte Carlo (MCMC) iterations to 100,000, with a burn-in of 50,000. The number of clusters was inferred based on the average linkage hierarchical clustering in the post-burn-in trace by optimizing the maximization of posterior expected adjusted Rand index criterion.

### Single-cell RNA sequencing and processing

scRNA-seq and data processing were carried out as previously described [24]. Briefly, cells isolated from human mRCC, PDX-mRCC, and paired PDX-pRCC tumors were subjected to single-cell capture and cDNA amplification using the C1™ Single-Cell Auto Prep System (Fluidigm, South San Francisco, CA, USA) with the SMARTer kit (Clontech, Mountain View, CA, USA). RNA-seq reads for single cells and bulk samples were generated using the HiSeq 2500 in the 100-bp paired-end mode and reads that only mapped to the mouse genome reference (mm10) were subsequently removed. Filtered reads were aligned to the human genome reference hg19 with the sample specific-splice junction using the 2-pass mode of STAR (Ver-2.4.0d) [59]. Transcripts per million (TPM) was quantified using RSEM (Ver-1.2.18) [60]. To filter out poor quality cells, we applied the critea of >1 M reads per cell, >60 % uniquely mapped rate, >35 % exonic region coverage rate, and >5000 detected genes. We considered TPM values greater than 1 to be reliable and only focused on genes that were detected in more than 10 % of a group between pRCC and paired lung metastasis. To identify differential and common expression signatures between pRCC and paired lung metastasis single cells compared to normal signals, we used expression profiles of normal kidney cortex from the GTEx portal (http://www.gtexportal.org/home/; transcript read counts V3) by converting to TPM values. Finally, we normalized sample-to-sample variation by applying a mean centroid.

### Estimation of activity for expression signatures and drug sensitivities

To understand the relative activation status for a pathway or signature across samples, we implemented the GSVA algorithm [61] in RNA-seq data. GSVA scores were estimated for given gene sets from the following sources: stromal signature, extracted from the ESTIMATE package [62]; ccRCC signature, taken from Jones et al. [63]; EMT-induced signature, taken from Taube et al. [64]; metastatic signature, taken from Jones et al. [63]; prognostic signature, taken from The Cancer Genome Atlas Research Network for ccRCC [25]; EGFR signaling, (Reactome, signaling by constitutively active EGFR); Src signaling, extracted from Gatza M.L. et al. [65]; mTOR signaling (Reactome, mTOR signaling); VEGFR signaling (PID, signaling events mediated by VEGFR1 and VEGFR2); RAF signaling, (Reactome, RAF activation); MEK signaling, (Reactome, MEK activation); c-Met signaling, (PID, signaling events mediated by c-Met); SCF-KIT signaling, (Reactome, signaling by SCF-KIT); PI3K/AKT signaling (Reactome, PI3K/AKT signaling in cancer); FGFR signaling (Reactome, signaling by FGFR); PDGFR signaling (PID, PDGF receptor signaling network). To evaluate whether an estimated gene set signature is significantly activated, we transformed the observed GSVA scores to binary scores. Gene sets with the same size as each original panel of genes were randomly generated with permutation (×1000) and computed for their GSVA scores. Assignment of the original GSVA scores as “activated” was determined with the cutoff values of averaged scores in the randomly selected gene sets.

In addition to estimation of activity status for signaling pathways, relevant targeting drug sensitivities were also predicted from expression profiles. Following a previous approach [30], cancer cell line expression data with measured drug response data from the Cancer Genome Project (CGP) [31] were used as a training set. After adjusting two independent sets of our expression data with the training set of solid tumor cell lines using ComBat [66], a ridge regression model was fitted for the training set with the given drugs that were simultaneously identified in our study. The lowest varying 20 % of genes were filtered out to focus on biological variability over technical variability. To evaluate the prediction sensitivity, leave-one-out-cross-validation (LOOCV) was applied with the total dataset. From this computation for a total of 10 drugs (BIBW2992 [Afatinib], Dasatinib, Gefitinib, Erlotinib, Temsirolimus, Pazopanib, Sunitinib, Sorafenib, AZD6244 [Selumetinib], and PF-02341066 [Crizotinib]), our data were tested to predict drug sensitivity and the distribution of estimates was transformed to Z-scores by dividing the averaged drug sensitivity estimates by the standard deviation for the difference. To determine the prediction accuracy of drug sensitivity estimates with measured drug sensitivity from high-throughput drug screening, nanomolar scaled IC_50_ values were also transformed to Z-scores.

### Functional network analysis

To identify discrete biological networks respectively enriched in pRCC and mRCC cells, differentially expressed genes (DEGs) were first defined with a criteria of the Benjamini–Hochberg corrected FDR <0.01 and fold changes of at least twofold. DEGs were separately applied to Gene Ontology (GO) category analysis using the ClueGO [67] plug-in within the Cytoscape framework [68]. The statistical significance for the over-represented pathways in the GO Biological Process category was estimated using Benjamini–Hochberg correction for multiple testing-controlled *P* values. Significantly enriched terms were functionally grouped based on kappa scores >0.3.

### Primary in vitro short-term culture

Xenograft tumor specimens were dissociated into single cells according to previously published protocols [69]. Dissociated PDX cells were cultured in neurobasal media-A supplemented with N2 (×1/2, Life Technologies, Carlsbad, CA, USA), B27 (×1/2, Gibco), basic fibroblast growth factor (20 ng/mL; R&D Systems, Minneapolis, MN, USA), epidermal growth factor (EGF, 20 ng/mL; R&D Systems), neuregulin 1 (10 ng/mL; R&D Systems), and insulin-like growth factor 1 (100 ng/mL; R&D Systems) and containing 10 % conditioned medium (CM) from human mesenchymal stem cells (MSCs). To generate the CM, MSCs were grown to 70 % confluency in plates with 10 % FBS L-DMEM and allowed to adhere overnight at 37 °C and 5 % CO_2_. The following day, the medium was replaced with serum-free culture medium and the cells were cultured for 2 days. The used medium was collected as MSC-CM, centrifuged to remove cell debris, and passed through a 0.45-μm filter. CM aliquots were frozen at −80 °C until use.

### In vitro drug sensitivity assay

Primary RCC PDX cells cultured under serum-free sphere culture conditions were seeded in 384-well plates at 500 cells per well. Two hours after plating, the cells were treated with a drug library in threefold and 10-point serial dilution series (n = 3 for each condition). After incubation at 37 °C in a 5 % CO_2_ humidified incubator for 6 days, cell viability was analyzed using an adenosine triphosphate monitoring system based on firefly luciferase (ATPliteTM 1step, PerkinElmer, Waltham, MA, USA). The drug library was composed of 20 targeted agents that were included in the clinical guidelines or in current clinical trials (gefitinib, erlotinib, lapatinib, afatinib, tivantinib, foretinib, crizotinib, selumetinib, temsirolimus, everolimus, cabozantinib, vandetanib, sunitinib, sorafenib, dovitinib, vemurafenib, BKM 120, pazopanib, nintedanib, and DAPT; all purchased from Selleckchem, Houston, TX, USA). The drugs were stored and diluted according to the manufacturer’s instructions. Test concentrations for each drug were empirically derived to produce a clinically relevant spectrum of drug activity. Half-maximal (50 %) inhibitory concentration values (IC_50_) were calculated as an average of triplicate experiments using the S+ Chip Analyzer (Samsung Electro-Mechanics Company, Ltd., Gyeonggi, Korea) [70].

For signal transduction assays under treatment with the targeted agents, primary cultured PDX cells were maintained overnight in serum-free sphere culture conditions without growth factors, incubated for 1 h with each inhibitor, and pulsed with original culture medium supplemented for 15 min. For immunoblotting, cells were lysed in RIPA lysis buffer supplemented with 1× phosphatase inhibitors (PhosStop; Roche Diagnostics, Basel, Switzerland) and a 1× protease inhibitor cocktail (Complete Mini; Roche Diagnostics). After centrifugation at 10,000 × *g* for 5 min, the supernatant was harvested and protein concentration was determined using a bicinchoninic acid protein assay kit (Thermo Scientific, Waltham, MA, USA). Equal amounts of protein were subjected to SDS-PAGE and transferred to polyvinylidene difluoride membranes (Whatman plc, Little Chalfont, UK). Membranes were blocked in 5 % skim milk or bovine serum albumin for 1 h at room temperature, incubated with the indicated primary antibodies overnight, and then with the appropriate secondary antibodies. Antibodies against p-EGFR (Tyr1068), p-Src (Tyr527), p-ERK, p-AKT (Ser473) (all purchased from Cell Signaling Technology, Danvers, MA, USA), and GAPDH (Santa Cruz Biotechnology, Santa Cruz, CA, USA) were used.

### Preparation of microfluidic drug screening device

The microfluidic drug screening device was made of polydimethylsiloxane (PDMS, Sylgard 184; Dow Corning, Corning, NY, USA) using a conventional softlithography process with 200 micron high SU-8 patterned silicon wafer (MicroChem, Westborough, MA, USA). The fabricated device was sterilized and bonded onto a cover glass to enclose the microchannels by oxygen plasma treatment (Femto Science, Somerset, NJ, USA). The device was then heated in an oven at 80 °C for 24 h to allow the surface to recover its hydrophobicity. The restored hydrophobicity of the microfluidic channel surface helps the injected extracellular matrix (ECM) form a stable interface with the side channels. In repeated measures analysis for drug sensitivity, the identical experimental condition was applied to the second measurement in PDX-mRCC cells.

### Three-dimensional cell culture and drug treatment

Collagen type 1 (3 mg/mL, rat tail; Corning) was used as an ECM scaffold to embed the cells. The collagen solution was prepared at pH 7 and a concentration of 2 mg/mL. This solution was diluted in a mixture of 10× phosphate-buffered saline (PBS; Gibco) and sterilized deionized water. The pH was adjusted using 0.5 N NaOH. Dissociated cells were suspended in the collagen solution at a density of 0.5 × 10^6^ cells/mL. The suspension was injected into a center channel of the device and allowed to gel by incubation at 37 °C and 5 % CO_2_ for 30 min. Details of the device preparation and gel filling procedure have been described previously [41]. To avoid cell attachment to the microfluidic channel surface, the device was inverted every 5 min. After gel formation, the side channels were filled with medium containing each drug candidate. The medium in the channel was refreshed every 24 h.

### Live/dead assay

Cell viability was quantified at 4 and 7 days of culture using the Live/Dead Viability Assay Kit (Molecular Probes, Invitrogen, Carlsbad, CA, USA) containing calcein AM and ethidium homodimer to identify live (green) and dead (red) cells, respectively. Cells in the microfluidic device were incubated at 37 °C with 5 % CO_2_ for 30 min, and then the staining solution was replaced with PBS. The cells were counted using ImageJ software (Image Processing and Analysis in Java, NIH, Bethesda, MD, USA). Cell viability was calculated as the number of live cells divided by the total cell number. Normalized cell viability was determined by dividing cell viability by the viability of cells cultured in pure medium condition.

### In vivo drug efficacy

BALB/c nude mice (female, 6–8 weeks old; Orient Bio) were used for drug efficacy studies. Animal experiments were conducted in accordance with the Institute for Laboratory Animal Research Guide for the Care and Use of Laboratory Animals and the following protocols were approved by the IRB at Samsung Medical Center (Seoul, Korea). Briefly, 2 × 10^5^ dissociated PDX-mRCC cells mixed 1:1 with Matrigel (BD Biosciences) were inoculated subcutaneously into the right flank of each mouse. Tumor diameters were measured with calipers twice a week and tumor volume in mm^3^ was calculated using the following formula: tumor volume = (l × w^2^)/2, where l is the longest diameter of the tumor and w is the shortest diameter of the tumor. Mice bearing established tumors (100–150 mm^3^) were randomly allocated to four groups (5 in each group): vehicle, afatinib at 20 mg/kg (p.o.), dasatinib 15 mg/kg (i.p.), or afatinib/dasatinib combination on a daily dosing regimen for up to 15 days. Mice in the four groups exhibited similar average tumor volumes and body weight. Throughout the study, the mice were weighed and tumor burden was monitored every 3 days. Mean tumor volumes were calculated, and growth curves were established as a function of time. The error bars indicate the value of the standard error of the mean (SEM). Tumors from each group were collected at the end of the experiment for further analysis.

### Immunohistochemical and TUNEL staining

Tumors were embedded in paraffin, sectioned at 5 μm, and stained with H&E. Paraffin-embedded tissue sections were deparaffinized and rehydrated. For immunochemical staining, heat-induced epitope retrieval was performed using a target retrieval solution (Dako, Glostrup, Denmark) for 5 min in a microwave. Slides were treated with 3 % hydrogen peroxide for 10 min to inactivate endogenous peroxidase and then blocked for 20 min at room temperature (RT) in blocking solution (5 % normal horse serum, 1 % normal goat serum, 0.1 % Triton-X 100 in 1× PBS). After blocking, the slides were incubated with primary antibodies, including mouse monoclonal antibody against p-ERK (Cell Signaling Technology) and human Ki-67 (BD Pharmingen). After washing, the slides were incubated with secondary antibodies for 1 h at RT, and counterstained with hematoxylin (Sigma-Aldrich, St. Louis, MO, USA). Apoptotic cells were identified by histologic analysis of DNA fragmentation in paraffin sections of the xenograft tumors. We performed terminal deoxynucleotide transferase-mediated dUTP nick end labeling (TUNEL) staining on the tumor sections using the DeadEnd™ Colorimetric TUNEL System (Promega, Madison, WI, USA). The proliferative or apoptotic index was calculated as a ratio of Ki-67-positive or TUNEL-positive cell number to total cell number in high-power (×400) fields.

### Statistical analysis

All values are expressed as the mean ± SEM. Comparisons between two groups were analyzed by Student’s *t*-test. One-way analysis of variance was applied for comparisons between more than two groups and to determine the statistical significance for the fitting model in the linear regression of two components. All *P* values were two-sided, and *P* <0.05 was considered statistically significant. For discovery of differentially expressed genes (DEGs) between pRCC and mRCC cells, we applied the Benjamini–Hochberg correction for multiple-testing. Statistically significant DEGs were regarded using the cutoff of FDR <0.01 and fold-change ≥2. Multiple regression analysis was applied to evaluate the transcriptomic heterogeneity across single cells, and to estimate the explanatory power of randomly selected single cells (with the given number by permutation × 1000) attributed to the expression profile of the bulk measurement by calculating the adjusted R-square. All data analyses were performed using SPSS statistical software, version 19.0 (SPSS, Inc., Chicago, IL, USA).

### Data access

RNA sequence and aCGH data have been deposited in the National Center for Biotechnology Information (NCBI) Gene Expression Omnibus (GEO) under accession number GSE73122. Whole-exome sequence data can be accessed at the NCBI Sequence Read Archive (SRA) with accession number SRP063388.

## Additional files

Additional file 1: Figure S1. Histologic and genetic similarity between parental tumor and PDX model. A Morphologic similarity between parental tumors and matched xenografts by hematoxylin and eosin (H&E) staining and bright-field observation for tumor tissues and PDX cells, respectively. B Tumor purity and ploidy of the PDX and parental tumors were computationally estimated using the ABSOLUTE algorithm. C Somatic single-nucleotide variants (SSNVs) of the PDX and parental tumors are compared in a *Venn diagram*. The number of SSNVs shared by all four tumors is colored *yellow*. D Changes in cellular prevalence of SSNVs are presented. SSNVs that were shared by all four tumors (n = 59) are highlighted by *yellow outlined circles* and *thick lines*. Non-silent SSNVs common in ccRCC (Additional file 2: Figure S2C) are denoted. E Somatic copy number alterations (SCNAs) along the autosomal chromosomes of the PDX and parental tumors were adjusted with estimated tumor purity and ploidy and presented as *heatmaps*. The order of tumors was determined by average linkage clustering in Euclidean distance similarity metrics. Common SCNAs of ccRCCs are identified in Additional file 2: Figure S2B. F Pearson correlation coefficients (r) of copy number profiles between samples are presented. (PDF 2.57 mb) Additional file 2: Figure S2. Comparative genetic profiles of paired pRCC and mRCC. A *Left*, variant allele frequencies (VAF) of somatic single-nucleotide variants (SSNVs) in pRCC and mRCC are presented as a *dot plot. Gray*, *orange*, and *blue* represent shared, pRCC-exclusive, and mRCC-exclusive SSNVs, respectively. *Center*, numbers of exclusive and shared SSNVs are shown in a *Venn diagram. Right*, *boxplots* demonstrating VAF distributions of exclusive and shared SSNVs of pRCC and mRCC. *Boxes* show 25th to 75th percentile with 10th and 90th percentile whiskers. Two-tailed Student’s *t*-test was applied to determine statistical significance of differences in VAF distribution. For a full list, see Additional file 3: Table S1. B Array comparative genomic hybridization (aCGH) copy number profiles of pRCC and mRCC. Break points of CGH probes were detected using the circular binary segmentation (CBS) algorithm. Regions of differential copy number between pRCC (*orange*) and mRCC (*blue*) are shadowed with arrows along the chromosomes. CBS-derived copy gains (*red*) and losses (*blue*) were defined using a log2 ratio cutoff of positive 0.25 and negative 0.25, respectively. C Somatic copy number alterations (SCNAs) and SSNVs of pRCC and mRCC were compared at the common SCNA and SSNV sites of clear cell RCC (ccRCC). Filtering criteria for SSNVs and SCNAs are indicated. Only non-silent SSNVs are annotated. (PDF 484 kb) Additional file 3: Table S1. Somatic mutations identified in tumor samples from whole-exome sequencing. Single-nucleotide variants (SNVs) were annotated by implementing SnpEff. Whole-exome sequencing data of paired patient blood was used to identify somatic variants in parental tumors and matched xenografts of pRCCs and mRCCs, which are shown in separate sheets. (XLSX 218 kb) Additional file 4: Figure S3. Clonal evolution of RCC during metastatic spread to lung. Gaussian kernel density estimates were applied in a post-burn-in of Markov Chain Monte Carlo (MCMC) traces in the PyClone algorithm. A Total SSNVs are annotated using the official gene symbol with chromosomal position and non-silent mutations are highlighted in *bold. Arrows* indicate underlying driver mutations in Additional file 2: Figure S2C. B Mean cellular prevalence of each SSNV (for full list, see Additional file 5: Table S2). Dominant subclones harboring SSNVs with higher cellular prevalence are ordered starting from the left. C Inferred phylogenetic evolutionary pattern between pRCC and mRCC. Branch and trunk lengths are proportional to the number of SSNVs. SSNVs and SCNAs that were significantly observed in ccRCC TCGA data (Additional file 2: Figure S2C) are denoted. (PDF 393 kb) Additional file 5: Table S2. Estimated subclonal frequencies of propagated somatic mutations in the evolutionary trajectory. Annotated information corresponding to somatic single-nucleotide variants (SSNVs) was taken from Additional file 3: Table S1. Mean cellular frequencies from a post-burn-in of 50,000 chains of MCMC analysis in PyClone are shown with subclonal information. (XLSX 15.3 kb) Additional file 6: Figure S4. Performance assessment of single-cell RNA-seq data. Histograms of A singlecell frequencies in the number of generated RNA-seq reads per cell, B uniquely mapped reads, C exonic regional coverage rate, and D detected number of genes per cell. *Dashed red lines* indicate the mean of the *x-axis* values. Using filtering criteria of >1 M reads per cell, >60 % uniquely mapped rate, >35 % exonic region coverage rate, and >5000 detected genes, two single cells were not included in subsequent analysis (for details, see Additional file 7: Table S3). (PDF 345 kb) Additional file 7: Table S3. Sequencing statistics for bulk cells and single-cell RNA-seq. Mapping information (to the hg19 genome reference) was obtained from log files after running STAR. RNA-SeQC was applied to generate a series of quality control metrics. (XLSX 21.4 kb) Additional file 8: Figure S5. Evaluation of the averaged expression levels of genes with their variations across single-cell populations. A, B Filtered genes expressed as log2 ratio of transcripts per million (TPM) + 1 were averaged and their standard deviations calculated across respective subgroups of single cells or the normal kidney cortex A, and across mixed subgroups B. The coefficient of variation (CV) is denoted and shown as the slope in the *dotted gray line*. Selected housekeeping genes are highlighted in *red* with their gene symbols. (PDF 1.45 mb) Additional file 9: Figure S6. Expression signatures of tumor cells compared to the normal kidney cortex. A, B Gene expressions of normal kidney cortexes were downloaded from the GTEx portal (n = 8, Ver.3). To identify outlier values in normal distribution, Z-scores were estimated and are shown in *radial* and *QQ-plots* for gene sets of the stromal A and ccRCC B signatures. (PDF 135 kb) Additional file 10: Figure S7. Transcriptomic similarity between primary tumor and PDX model. A, B *Scatter plots* show reciprocal similarities of global gene expression between bulk samples A, and between averaged single cells and bulk samples B. *Black dotted line* is the x = y line with correlation coefficients (Pearson and Spearman r) for linear fit. The *x-y axes* represent log2 ratio of TPM + 1. C Explanatory power (adjusted R-square) of gene expression of single cells to those of bulk cell population was estimated by multiple regression analysis with a randomly selected given number of cells with permutation (×1000). *Boxes* show 25th to 75th percentile with 10th and 90th percentile whiskers. Median values within the boxes are represented as *diamond symbols* and connected in *lines between boxes. Dotted vertical lines* indicate the explanatory power with the total single cells. (PDF 548 kb) Additional file 11: Figure S8. Gene set activation analysis for the epithelial epithelial-mesenchymal transition (EMT)-induced signature. A Positions of each *dot* and *ellipse* in Fig. 1d were fixed, and then each *dot* was colored (*main panel*) and indicated as *drop lines* (*top and right panels*) according to the estimated activation status in the EMT-induced signature. B *Boxplots* show overall reciprocal differences in the expression signatures across normal kidney cortex, bulk cells of each population, and single cells. *Boxes* show 25th to 75th percentile with 10th and 90th percentile whiskers. ****P* <0.001, two-tailed Student’s *t*-test. (PDF 124 kb) Additional file 12: Figure S9. Functionally grouped networks based on analysis of differentially expressed genes between pRCC and mRCC cells. A A volcano plot of gene expression (log2 ratio of TPM + 1) contrasting pRCC cells (n = 46) versus mRCC cells (n = 36). The *vertical dotted line* indicates the cutoff of the false discovery rate (FDR <0.01), which was adjusted using the Benjamini–Hochberg correction for multiple-testing. The *horizontal dotted lines* indicate the cutoff for a significant fold change (≥twofold). *Colors* indicate significantly upregulated genes in pRCC cells (*red*) or in mRCC cells (*blue*). B Visualization of enriched biological networks with upregulated genes in pRCC cells (*top*) or in mRCC cells (*bottom*) by ClueGO analysis with annotation of Gene Ontology. Term enrichment significance is determined using two-sided hypergeometric test with the Benjamini–Hochberg correction, and represented by node size. Genes that are shared between two gene ontology terms generate link lines. The most prominent gene ontology term for each functional group is highlighted in a larger font size with the text color identical to the relevant group. (PDF 1.63 mb) Additional file 13: Figure S10. Activity of druggable pathways of pRCC and mRCC cells in the PCA plot. The plots show heterogeneous activated status of targetable signaling pathways at single-cell resolution (in the *scatter plot* with *colored dots*) compared to bulk cells. Positions of each dot and ellipses were derived from the PCA analysis in Fig. 1d. Colors of *dots* (*main panel*) and *drop lines* (*top and right panels*) represent the relative activation status of targetable signaling pathways. Gene Set Variation Analysis (GSVA) scores were normalized to normal kidney tissue expression profiles (shown in the upper *heatmap*). (PDF 316 kb) Additional file 14: Table S4. Results of drug screening for pRCC and mRCC tumors. Summarized list of drugs used in the screening and calculated nanomolar values of IC_50_. For repeated measures analysis, mRCC cells were tested twice. (PDF 94.2 kb) Additional file 15: Figure S11. Repeated measurement of high-throughput drug screening. The variance in the repeated measures analysis for drug sensitivity was evaluated in PDX-mRCC cells with the identical panel of drugs. The linear regression line (*black*) fitted on measured IC_50_ in duplicate is represented with 95 % confidence intervals (*gray*) over a theoretical regression line (*white diagonal*). The strength of the linear regression and its statistical significance was determined by Pearson’s correlation coefficient (*r*) and one-way ANOVA test, respectively. (PDF 279 kb) Additional file 16: Figure S12. Prediction of drug sensitivity across single-cell populations. A Drug sensitivity was predicted by the ridge regression model using a training set of publicly available cancer cell line expression data with measured IC_50_ data for each drug. Estimated values were transformed to Z-scores across samples. *Boxes* show 25th to 75th percentile with 10th and 90th percentile whiskers. Differences between groups were determined by two-tailed Student’s *t*-test. **P* <0.05, ***P* <0.01, ****P* <0.001. B Correlation of drug sensitivity with the relevant signaling pathways to be targeted. Uniform axis ranges were applied to all plots: *x-axis* of GSVA scores, −0.5 to 0.5; *y-axis* of Z-scores, −3 to 2. Linear regression was applied to estimate Pearson’s correlation coefficient (*r*), with 95 % confidence as shown in *thicker light gray curves*. The statistical significance of the regression was determined by one-way ANOVA test. (PDF 1.33 mb) Additional file 17: Figure S13. Recapitulation of poor response to pazopanib and everolimus in the patient through in vitro drug efficacy testing in 3D ECM scaffold system. In contrast to susceptibility to afatinib or dasatinib at a dose concentration of 0.5 μM (Fig. 3d), we did not detect significant anticancer activity of pazopanib or everolimus, even at higher dose concentrations. Results are presented as mean values ± SEM. (PDF 61.1 kb) Additional file 18: Figure S14. Correlation of drug sensitivity with the targeted pathways in classified subpopulations. The classified single-cell subpopulations shown in Fig. 4 were evaluated for correlation between drug sensitivity and the relevant signaling pathways to be targeted. Uniform axis ranges were applied to all plots: *x-axis* of GSVA scores, −0.5 to 0.5; *y-axis* of Z-scores, −3 to 2. Linear regression was applied to estimate Pearson’s correlation coefficient (*r*), with 95 % confidence as shown in *thicker light gray curves*. The statistical significance of the regression was determined by one-way ANOVA test. Although the absolute correlation coefficient (*r*) was mostly high across all comparisons, some showed an insignificant correlation due to the small size of samples involved in the statistical test, compared to the overall significant correlation in unclassified single-cell populations (Fig. 3). (PDF 878 kb)
