# Supplementary figures and images for: Application of single-cell RNA sequencing in optimizing a combinatorial therapeutic strategy in metastatic renal cell carcinoma
Source: Genome Biol. 2016 Apr 29;17:80. doi: 10.1186/s13059-016-0945-9 (PMC4852434; doi:10.1186/s13059-016-0945-9)

Figure S1

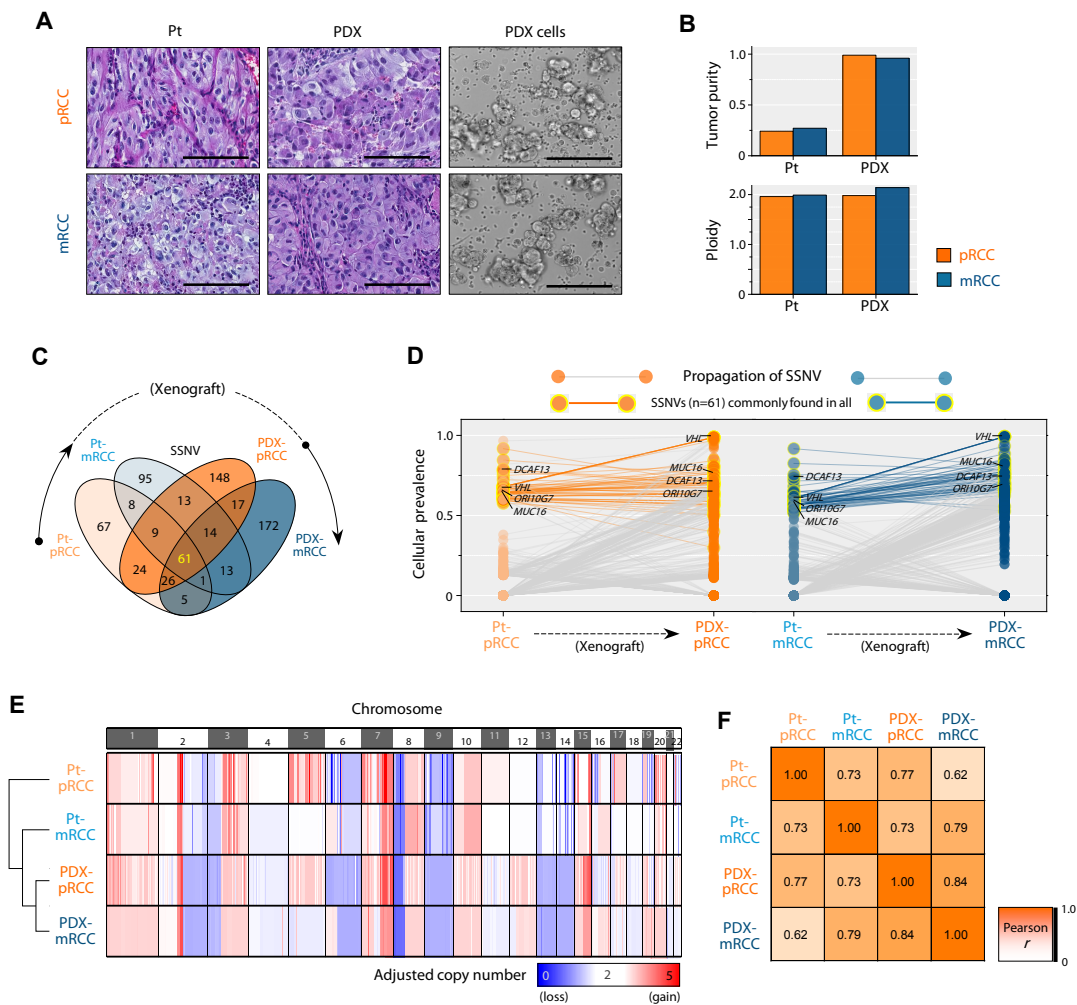

Supplement: Additional file 1: Figure S1. — Histologic and genetic similarity between parental tumor and PDX model. A Morphologic similarity between parental tumors and matched xenografts by hematoxylin and eosin (H&E) staining and bright-field observation for tumor tissues and PDX cells, respectively. B Tumor purity and ploidy of the PDX and parental tumors were computationally estimated using the ABSOLUTE algorithm. C Somatic single-nucleotide variants (SSNVs) of the PDX and parental tumors are compared in a Venn diagram. The number of SSNVs shared by all four tumors is colored yellow. D Changes in cellular prevalence of SSNVs are presented. SSNVs that were shared by all four tumors (n = 59) are highlighted by yellow outlined circles and thick lines. Non-silent SSNVs common in ccRCC (Additional file 2: Figure S2C) are denoted. E Somatic copy number alterations (SCNAs) along the autosomal chromosomes of the PDX and parental tumors were adjusted with estimated tumor purity and ploidy and presented as heatmaps. The order of tumors was determined by average linkage clustering in Euclidean distance similarity metrics. Common SCNAs of ccRCCs are identified in Additional file 2: Figure S2B. F Pearson correlation coefficients (r) of copy number profiles between samples are presented. (PDF 2.57 mb) [file 13059_2016_945_MOESM1_ESM.pdf]

Figure S2

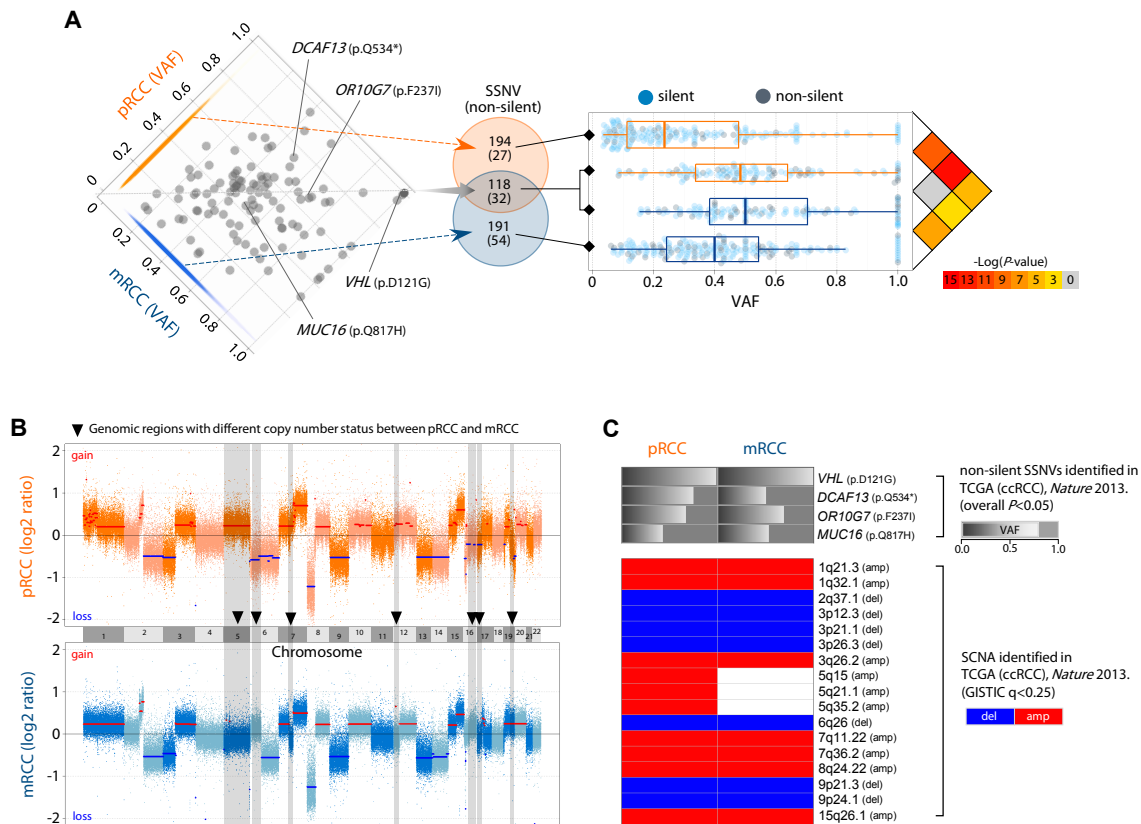

Supplement: Additional file 2: Figure S2. — Comparative genetic profiles of paired pRCC and mRCC. A Left, variant allele frequencies (VAF) of somatic single-nucleotide variants (SSNVs) in pRCC and mRCC are presented as a dot plot. Gray, orange, and blue represent shared, pRCC-exclusive, and mRCC-exclusive SSNVs, respectively. Center, numbers of exclusive and shared SSNVs are shown in a Venn diagram. Right, boxplots demonstrating VAF distributions of exclusive and shared SSNVs of pRCC and mRCC. Boxes show 25th to 75th percentile with 10th and 90th percentile whiskers. Two-tailed Student’s t-test was applied to determine statistical significance of differences in VAF distribution. For a full list, see Additional file 3: Table S1. B Array comparative genomic hybridization (aCGH) copy number profiles of pRCC and mRCC. Break points of CGH probes were detected using the circular binary segmentation (CBS) algorithm. Regions of differential copy number between pRCC (orange) and mRCC (blue) are shadowed with arrows along the chromosomes. CBS-derived copy gains (red) and losses (blue) were defined using a log2 ratio cutoff of positive 0.25 and negative 0.25, respectively. C Somatic copy number alterations (SCNAs) and SSNVs of pRCC and mRCC were compared at the common SCNA and SSNV sites of clear cell RCC (ccRCC). Filtering criteria for SSNVs and SCNAs are indicated. Only non-silent SSNVs are annotated. (PDF 484 kb) [file 13059_2016_945_MOESM2_ESM.pdf]

**Figure S4**

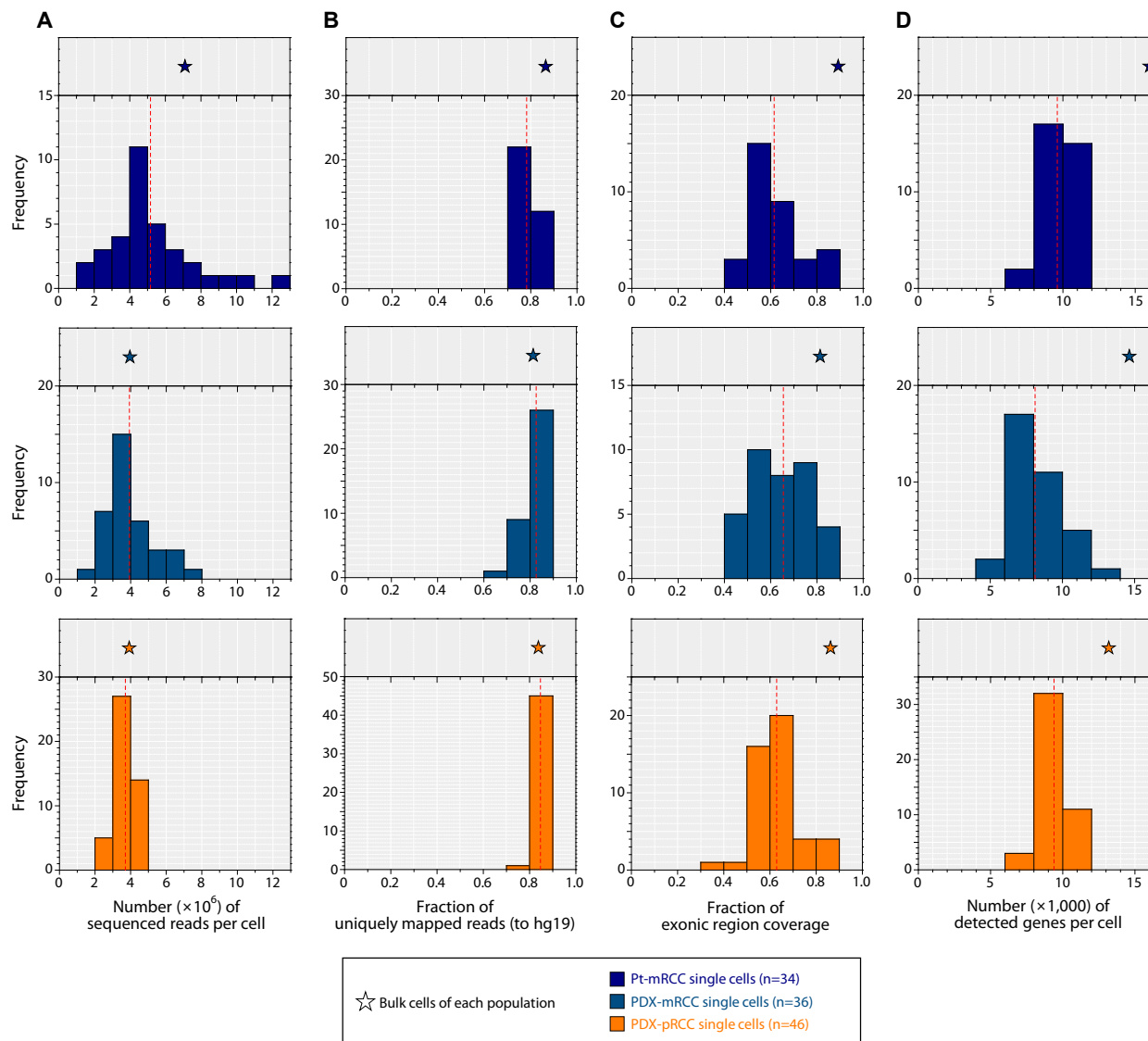

Supplement: Additional file 6: Figure S4. — Performance assessment of single-cell RNA-seq data. Histograms of A singlecell frequencies in the number of generated RNA-seq reads per cell, B uniquely mapped reads, C exonic regional coverage rate, and D detected number of genes per cell. Dashed red lines indicate the mean of the x-axis values. Using filtering criteria of >1 M reads per cell, >60 % uniquely mapped rate, >35 % exonic region coverage rate, and >5000 detected genes, two single cells were not included in subsequent analysis (for details, see Additional file 7: Table S3). (PDF 345 kb) [file 13059_2016_945_MOESM6_ESM.pdf]

**Figure S5**

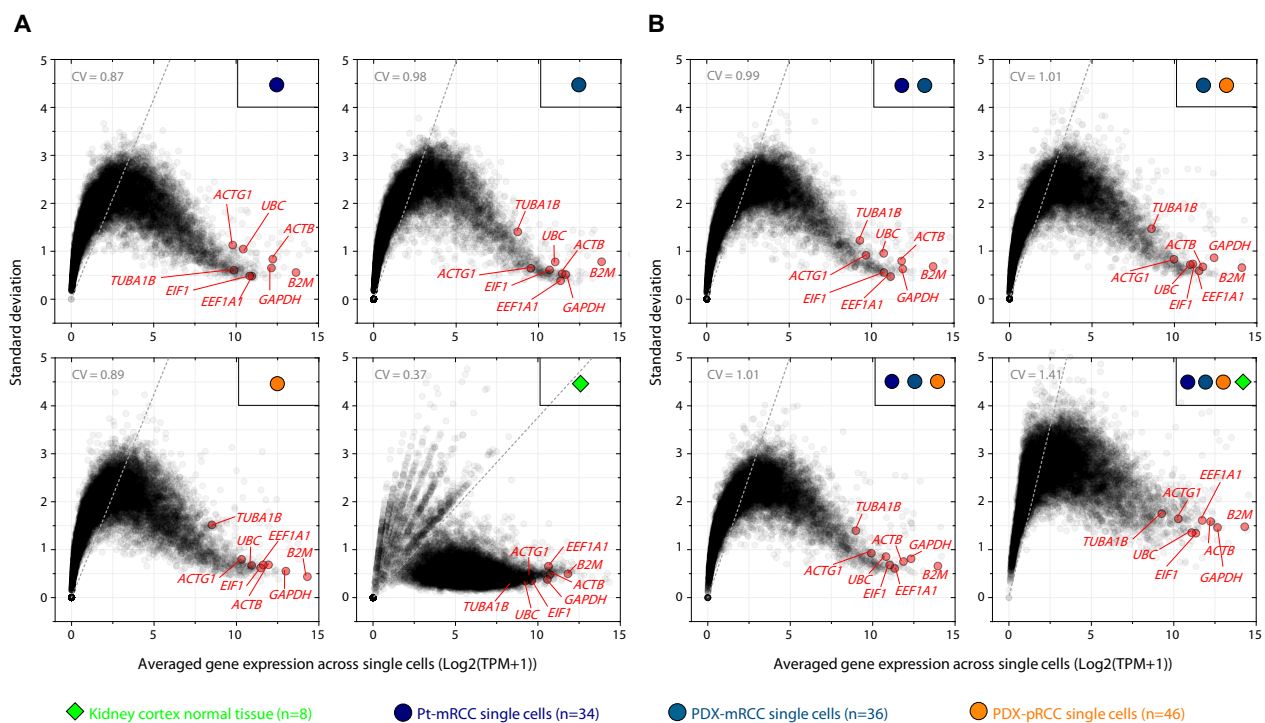

Supplement: Additional file 8: Figure S5. — Evaluation of the averaged expression levels of genes with their variations across single-cell populations. A, B Filtered genes expressed as log2 ratio of transcripts per million (TPM) + 1 were averaged and their standard deviations calculated across respective subgroups of single cells or the normal kidney cortex A, and across mixed subgroups B. The coefficient of variation (CV) is denoted and shown as the slope in the dotted gray line. Selected housekeeping genes are highlighted in red with their gene symbols. (PDF 1.45 mb) [file 13059_2016_945_MOESM8_ESM.pdf]

Figure S6

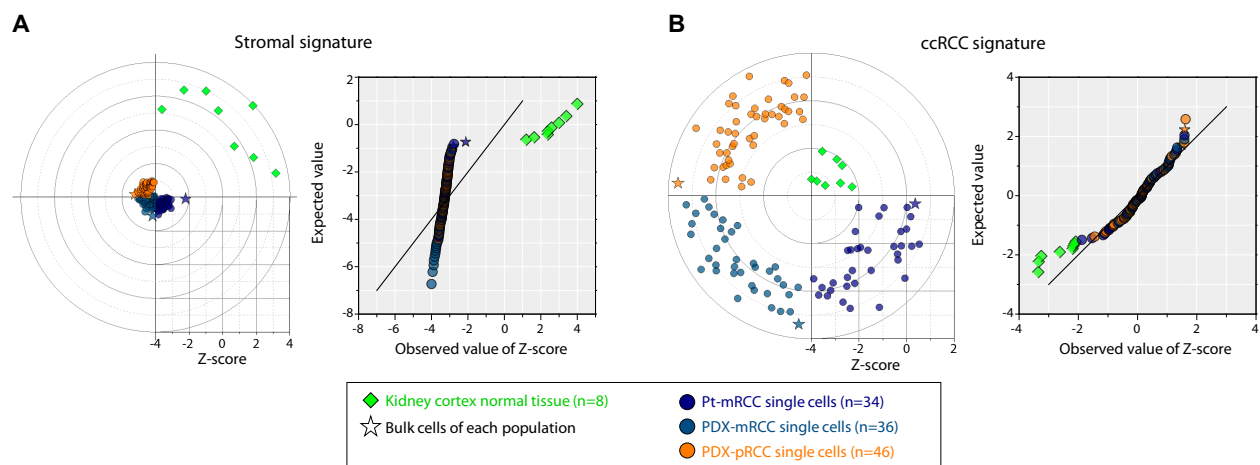

Supplement: Additional file 9: Figure S6. — Expression signatures of tumor cells compared to the normal kidney cortex. A, B Gene expressions of normal kidney cortexes were downloaded from the GTEx portal (n = 8, Ver.3). To identify outlier values in normal distribution, Z-scores were estimated and are shown in radial and QQ-plots for gene sets of the stromal A and ccRCC B signatures. (PDF 135 kb) [file 13059_2016_945_MOESM9_ESM.pdf]

### Figure S7

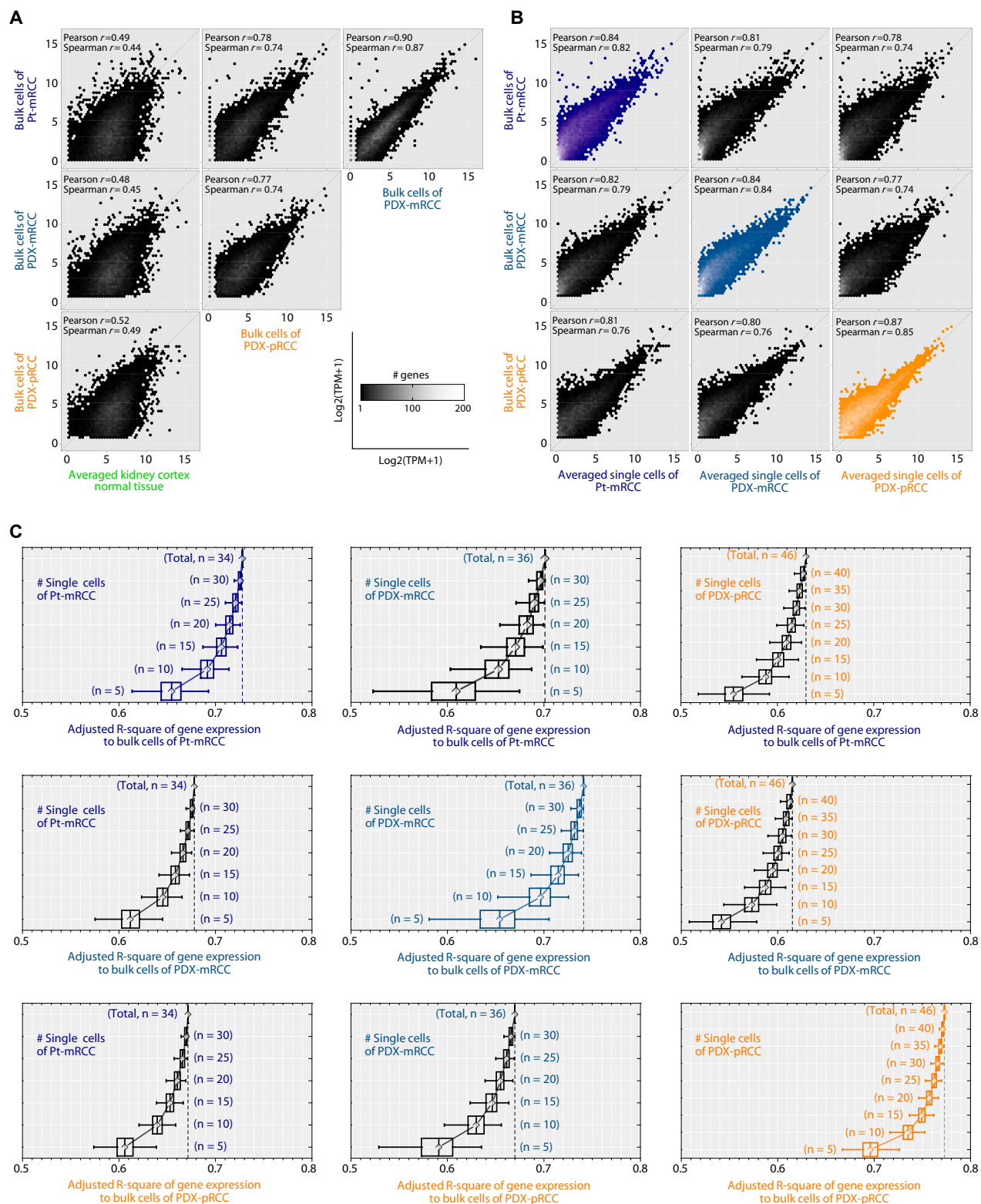

Supplement: Additional file 10: Figure S7. — Transcriptomic similarity between primary tumor and PDX model. A, B Scatter plots show reciprocal similarities of global gene expression between bulk samples A, and between averaged single cells and bulk samples B. Black dotted line is the x = y line with correlation coefficients (Pearson and Spearman r) for linear fit. The x-y axes represent log2 ratio of TPM + 1. C Explanatory power (adjusted R-square) of gene expression of single cells to those of bulk cell population was estimated by multiple regression analysis with a randomly selected given number of cells with permutation (×1000). Boxes show 25th to 75th percentile with 10th and 90th percentile whiskers. Median values within the boxes are represented as diamond symbols and connected in lines between boxes. Dotted vertical lines indicate the explanatory power with the total single cells. (PDF 548 kb) [file 13059_2016_945_MOESM10_ESM.pdf]

**Figure S8**

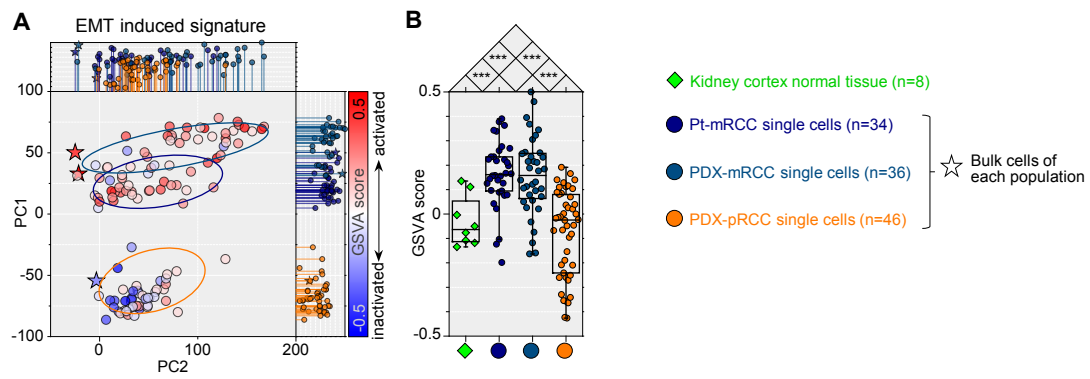

Supplement: Additional file 11: Figure S8. — Gene set activation analysis for the epithelial epithelial-mesenchymal transition (EMT)-induced signature. A Positions of each dot and ellipse in Fig. 1d were fixed, and then each dot was colored (main panel) and indicated as drop lines (top and right panels) according to the estimated activation status in the EMT-induced signature. B Boxplots show overall reciprocal differences in the expression signatures across normal kidney cortex, bulk cells of each population, and single cells. Boxes show 25th to 75th percentile with 10th and 90th percentile whiskers. ***P <0.001, two-tailed Student’s t-test. (PDF 124 kb) [file 13059_2016_945_MOESM11_ESM.pdf]

**Figure S9**

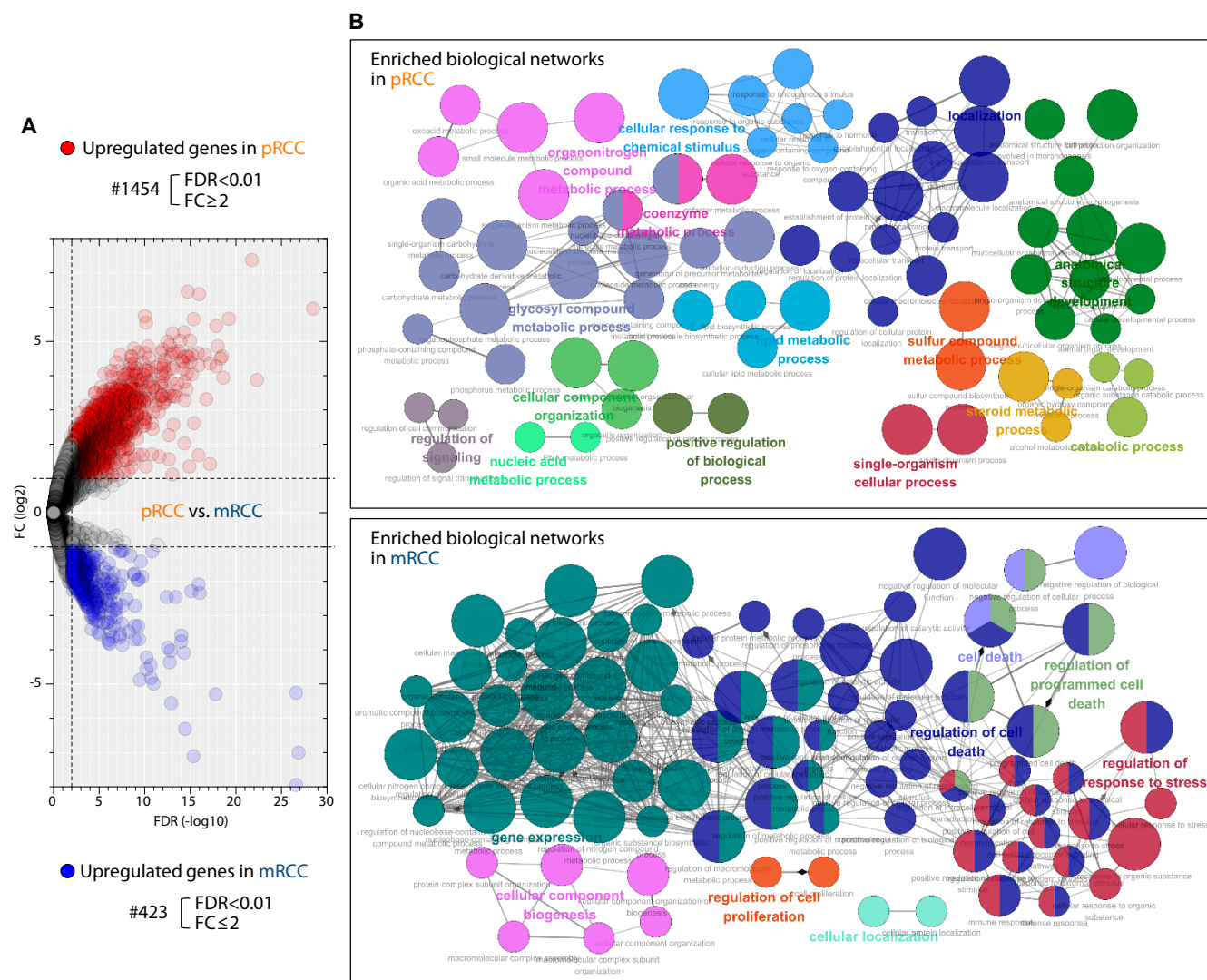

Supplement: Additional file 12: Figure S9. — Functionally grouped networks based on analysis of differentially expressed genes between pRCC and mRCC cells. A A volcano plot of gene expression (log2 ratio of TPM + 1) contrasting pRCC cells (n = 46) versus mRCC cells (n = 36). The vertical dotted line indicates the cutoff of the false discovery rate (FDR <0.01), which was adjusted using the Benjamini–Hochberg correction for multiple-testing. The horizontal dotted lines indicate the cutoff for a significant fold change (≥twofold). Colors indicate significantly upregulated genes in pRCC cells (red) or in mRCC cells (blue). B Visualization of enriched biological networks with upregulated genes in pRCC cells (top) or in mRCC cells (bottom) by ClueGO analysis with annotation of Gene Ontology. Term enrichment significance is determined using two-sided hypergeometric test with the Benjamini–Hochberg correction, and represented by node size. Genes that are shared between two gene ontology terms generate link lines. The most prominent gene ontology term for each functional group is highlighted in a larger font size with the text color identical to the relevant group. (PDF 1.63 mb) [file 13059_2016_945_MOESM12_ESM.pdf]

**Figure S10**

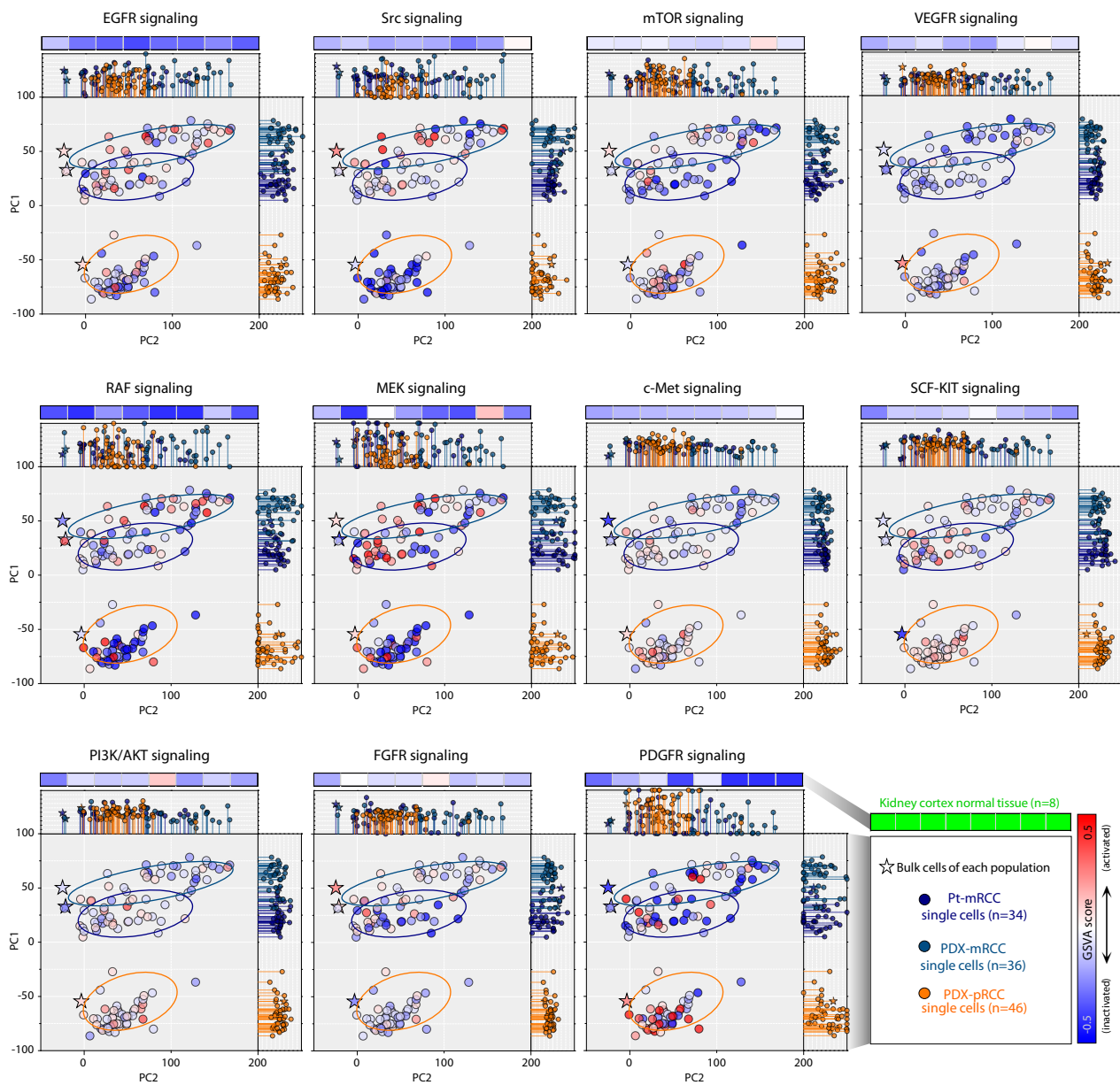

Supplement: Additional file 13: Figure S10. — Activity of druggable pathways of pRCC and mRCC cells in the PCA plot. The plots show heterogeneous activated status of targetable signaling pathways at single-cell resolution (in the scatter plot with colored dots) compared to bulk cells. Positions of each dot and ellipses were derived from the PCA analysis in Fig. 1d. Colors of dots (main panel) and drop lines (top and right panels) represent the relative activation status of targetable signaling pathways. Gene Set Variation Analysis (GSVA) scores were normalized to normal kidney tissue expression profiles (shown in the upper heatmap). (PDF 316 kb) [file 13059_2016_945_MOESM13_ESM.pdf]

**Figure S11**

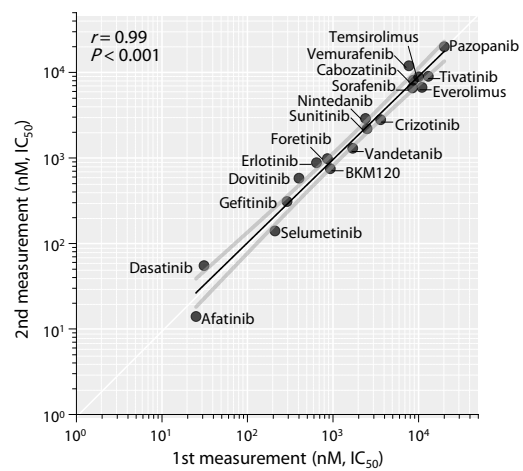

Supplement: Additional file 15: Figure S11. — Repeated measurement of high-throughput drug screening. The variance in the repeated measures analysis for drug sensitivity was evaluated in PDX-mRCC cells with the identical panel of drugs. The linear regression line (black) fitted on measured IC50 in duplicate is represented with 95 % confidence intervals (gray) over a theoretical regression line (white diagonal). The strength of the linear regression and its statistical significance was determined by Pearson’s correlation coefficient (r) and one-way ANOVA test, respectively. (PDF 279 kb) [file 13059_2016_945_MOESM15_ESM.pdf]

Figure S12

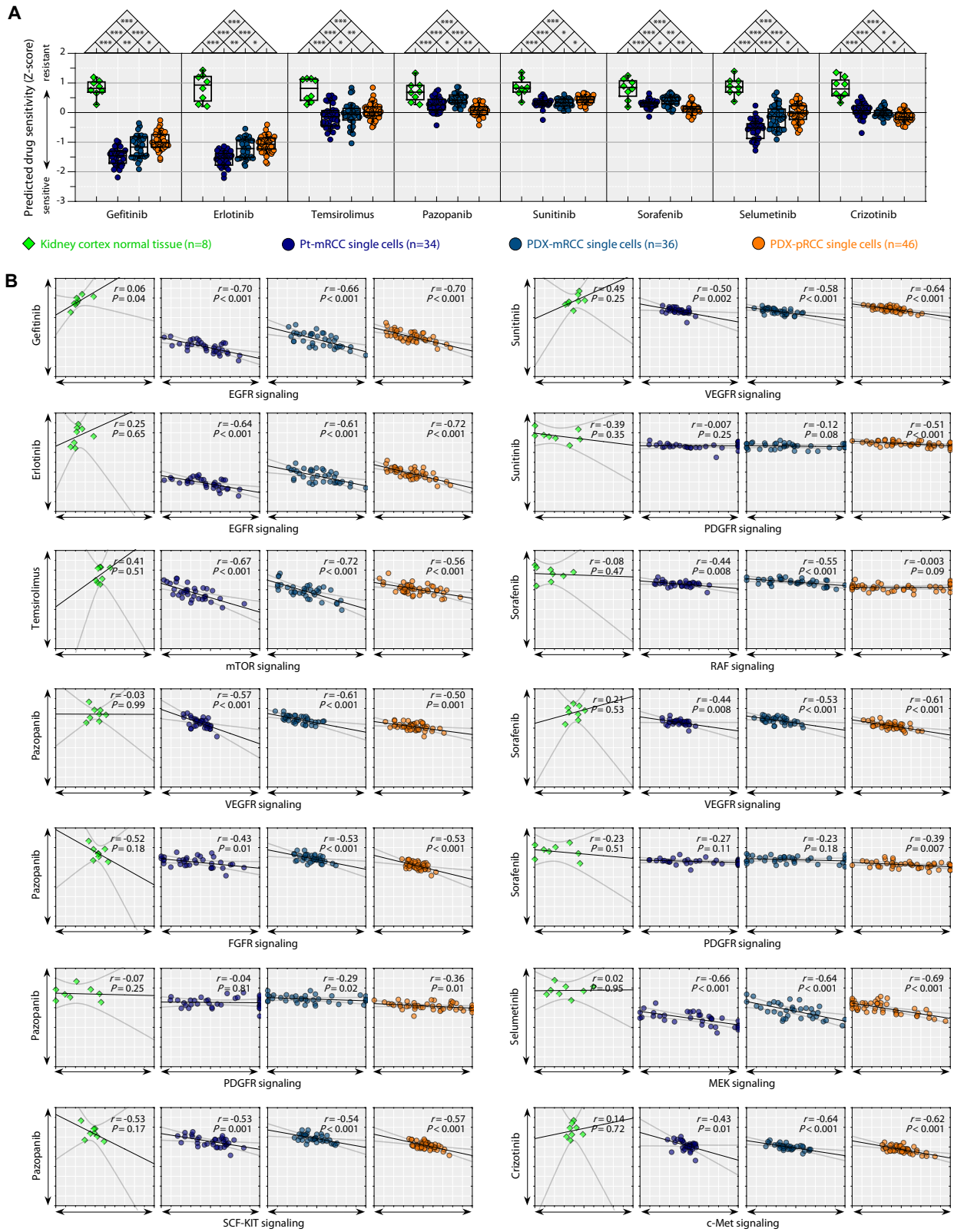

Supplement: Additional file 16: Figure S12. — Prediction of drug sensitivity across single-cell populations. A Drug sensitivity was predicted by the ridge regression model using a training set of publicly available cancer cell line expression data with measured IC50 data for each drug. Estimated values were transformed to Z-scores across samples. Boxes show 25th to 75th percentile with 10th and 90th percentile whiskers. Differences between groups were determined by two-tailed Student’s t-test. *P <0.05, **P <0.01, ***P <0.001. B Correlation of drug sensitivity with the relevant signaling pathways to be targeted. Uniform axis ranges were applied to all plots: x-axis of GSVA scores, −0.5 to 0.5; y-axis of Z-scores, −3 to 2. Linear regression was applied to estimate Pearson’s correlation coefficient (r), with 95 % confidence as shown in thicker light gray curves. The statistical significance of the regression was determined by one-way ANOVA test. (PDF 1.33 mb) [file 13059_2016_945_MOESM16_ESM.pdf]

**Figure S13**

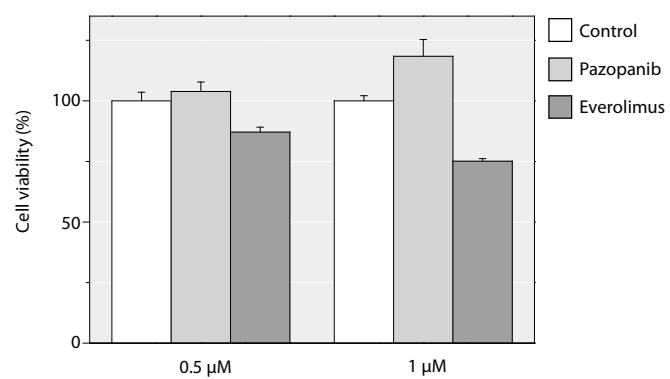

Supplement: Additional file 17: Figure S13. — Recapitulation of poor response to pazopanib and everolimus in the patient through in vitro drug efficacy testing in 3D ECM scaffold system. In contrast to susceptibility to afatinib or dasatinib at a dose concentration of 0.5 μM (Fig. 3d), we did not detect significant anticancer activity of pazopanib or everolimus, even at higher dose concentrations. Results are presented as mean values ± SEM. (PDF 61.1 kb) [file 13059_2016_945_MOESM17_ESM.pdf]

**Figure S14**

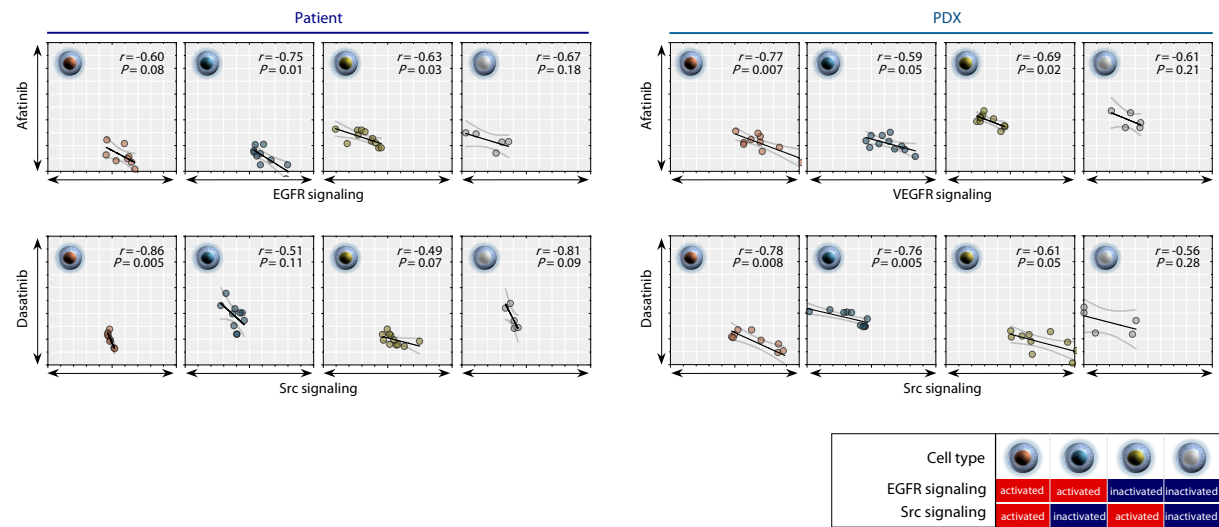

Supplement: Additional file 18: Figure S14. — Correlation of drug sensitivity with the targeted pathways in classified subpopulations. The classified single-cell subpopulations shown in Fig. 4 were evaluated for correlation between drug sensitivity and the relevant signaling pathways to be targeted. Uniform axis ranges were applied to all plots: x-axis of GSVA scores, −0.5 to 0.5; y-axis of Z-scores, −3 to 2. Linear regression was applied to estimate Pearson’s correlation coefficient (r), with 95 % confidence as shown in thicker light gray curves. The statistical significance of the regression was determined by one-way ANOVA test. Although the absolute correlation coefficient (r) was mostly high across all comparisons, some showed an insignificant correlation due to the small size of samples involved in the statistical test, compared to the overall significant correlation in unclassified single-cell populations (Fig. 3). (PDF 878 kb) [file 13059_2016_945_MOESM18_ESM.pdf]
