# Supplementary material for: Application of single-cell RNA sequencing in optimizing a combinatorial therapeutic strategy in metastatic renal cell carcinoma
Source: Genome Biol. 2016 Apr 29;17:80. doi: 10.1186/s13059-016-0945-9 (PMC4852434; doi:10.1186/s13059-016-0945-9)
Supplement: Additional file 4: Figure S3. — Clonal evolution of RCC during metastatic spread to lung. Gaussian kernel density estimates were applied in a post-burn-in of Markov Chain Monte Carlo (MCMC) traces in the PyClone algorithm. A Total SSNVs are annotated using the official gene symbol with chromosomal position and non-silent mutations are highlighted in bold. Arrows indicate underlying driver mutations in Additional file 2: Figure S2C. B Mean cellular prevalence of each SSNV (for full list, see Additional file 5: Table S2). Dominant subclones harboring SSNVs with higher cellular prevalence are ordered starting from the left. C Inferred phylogenetic evolutionary pattern between pRCC and mRCC. Branch and trunk lengths are proportional to the number of SSNVs. SSNVs and SCNAs that were significantly observed in ccRCC TCGA data (Additional file 2: Figure S2C) are denoted. (PDF 393 kb) [file 13059_2016_945_MOESM4_ESM.pdf]

Figure S3

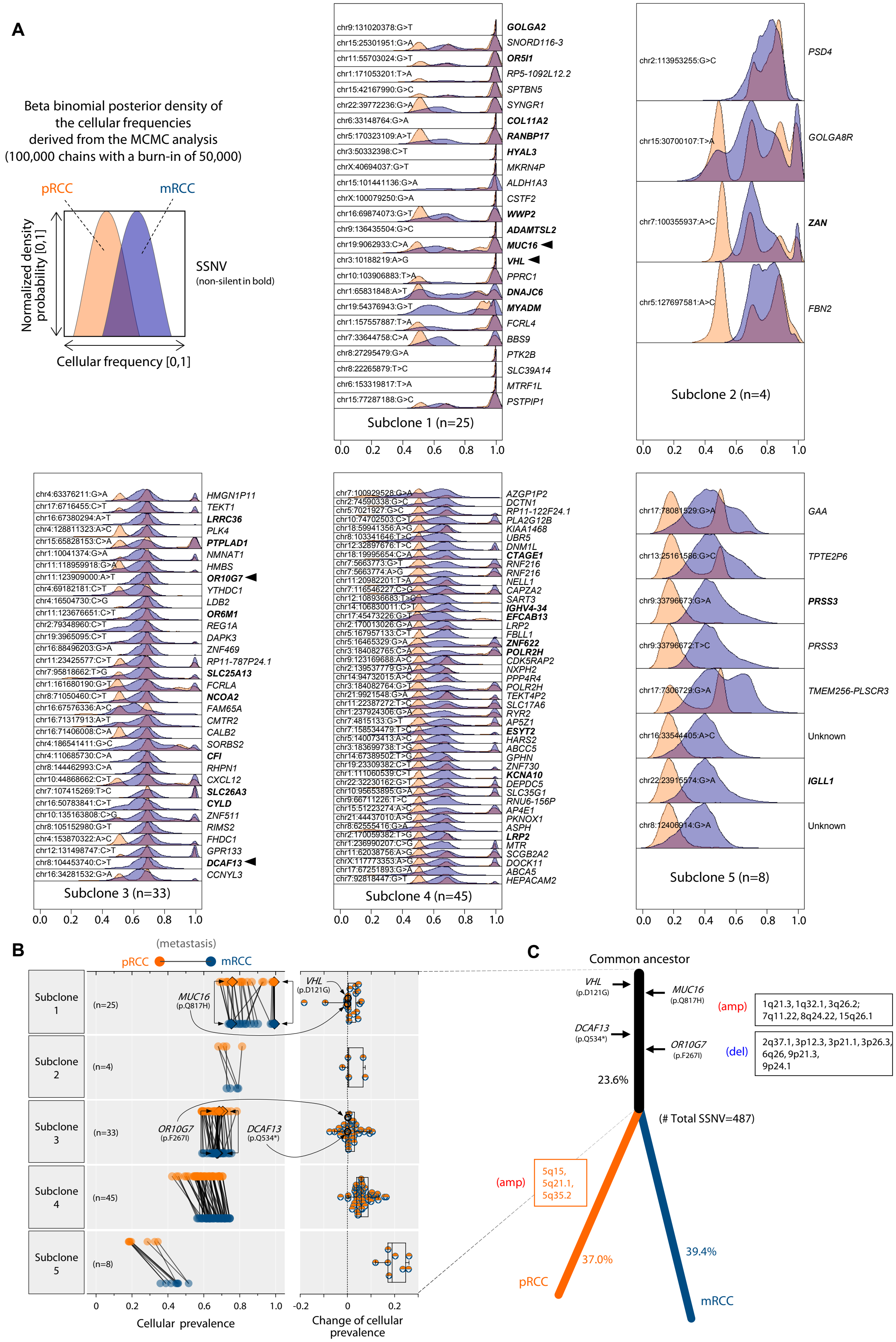

A
Beta binomial posterior density of
the cellular frequencies
derived from the MCMC analysis
(100,000 chains with a burn-in of 50,000)
pRCC
mRCC
Normalized density probability [0,1]
SSNV
(non-silent in bold)
Cellular frequency [0,1]
GOLGA2
SNORD116-3
OR5I1
RP5-1092L12.2
SPTBN5
SYNGR1
COL11A2
RANBP17
HYAL3
MKRN4P
ALDH1A3
CSTF2
WWP2
ADAMTSL2
MUC16
VHL
PPRC1
DNAJC6
MYADM
FCRL4
BBS9
PTK2B
SLC39A14
MTRF1L
PSTPIP1
Subclone 1 (n=25)
PSD4
GOLGA8R
ZAN
FBN2
Subclone 2 (n=4)
HMGN1P11
TEKT1
LRRC36
PLK4
PTPLAD1
NMNAT1
HMBS
OR10G7
YTHDC1
LDB2
OR6M1
REG1A
DAPK3
ZNF469
RP11-787P24.1
SLC25A13
FCRLA
NCOA2
FAM65A
CMTR2
CALB2
SORBS2
CFI
RHPN1
CXCL12
SLC26A3
CYLD
ZNF511
RIMS2
FHDC1
GPR133
DCAF13
CCNYL3
Subclone 3 (n=33)
Subclone 4 (n=45)
GAA
TPTE2P6
PRSS3
PRSS3
TMEM256-PLSCR3
Unknown
IGLL1
Unknown
Subclone 5 (n=8)
0.0 0.2 0.4 0.6 0.8 1.0
B
(metastasis)
pRCC
mRCC
Subclone 1 (n=25)
Subclone 2 (n=4)
Subclone 3 (n=33)
Subclone 4 (n=45)
Subclone 5 (n=8)
MUC16 (p.Q817H)
VHL (p.D121G)
OR10G7 (p.F267I)
DCAF13 (p.Q534*)
Cellular prevalence
Change of cellular prevalence
C
Common ancestor
VHL (p.D121G)
MUC16 (p.Q817H)
DCAF13 (p.Q534*)
OR10G7 (p.F267I)
(amp)
1q21.3, 1q32.1, 3q26.2; 7q11.22, 8q24.22, 15q26.1
(del)
2q37.1, 3p12.3, 3p21.1, 3p26.3, 6q26, 9p21.3, 9p24.1
23.6%
(# Total SSNV=487)
(amp)
5q15, 5q21.1, 5q35.2
37.0%
39.4%
pRCC
mRCC
