# Supplementary material for: Application of single-cell RNA sequencing in optimizing a combinatorial therapeutic strategy in metastatic renal cell carcinoma
Source: Genome Biol. 2016 Apr 29;17:80. doi: 10.1186/s13059-016-0945-9 (PMC4852434; doi:10.1186/s13059-016-0945-9)
Supplement: Additional file 14: Table S4. — Results of drug screening for pRCC and mRCC tumors. Summarized list of drugs used in the screening and calculated nanomolar values of IC50. For repeated measures analysis, mRCC cells were tested twice. (PDF 94.2 kb) [file 13059_2016_945_MOESM14_ESM.pdf]

Drug	Target	pRCC	mRCC (1st)	mRCC (2nd)
Gefitinib	EGFR	6400	290	310
Erlotinib	EGFR	>20000	640	880
Afatinib	EGFR/HER2	650	25	14
Tivantinib	C-Met	520	13000	9000
Foretinib	C-Met/VEGFR2	200	860	980
Crizotinib	C-Met/ALK	1200	3600	2800
Selumetinib	MEK	2400	210	140
Vemufafenib	B-Raf (V600E)	2800	7700	12000
Temsirolimus	mTOR	9500	10000	8900
Everolimus	mTOR	10000	11000	6700
BKM120	PI3K	500	930	750
Cabozatinib	VEGFR2	3800	8700	8100
Vandetanib	VEGFR2	1100	1700	1300
Sunitinib	VEGFR2/PDGFRβ	2200	2500	2200
Sorafenib	Raf-1/B-Raf/VEGFR2/PDGFRβ	4500	8500	6600
Pazopanib	VEGFR1-3/PDGFR/FGFR/c-Kit/c-Fms	5100	> 20000	> 20000
Nintedanib	VEGFR1-3/FGFR1-3/PDGFRα/β	1000	2400	2900
Dovitinib	FLT3/c-Kit/FGFR1/3/VEGFR1-4	1200	400	580
Dasatinib	Src, Abl	190	31	55
